# Genome-wide analysis and functional validation reveal the role of late embryogenesis abundant genes in strawberry (*Fragaria* × *ananassa*) fruit ripening

**DOI:** 10.1186/s12864-024-10085-9

**Published:** 2024-03-01

**Authors:** Yuanxiu Lin, Musha She, Mantong Zhao, Hong Yu, Wenfei Xiao, Yunting Zhang, Mengyao Li, Qing Chen, Yong Zhang, Yan Wang, Wen He, Xiaorong Wang, Haoru Tang, Ya Luo

**Affiliations:** 1 College of Horticulture, Sichuan Agricultural University, Chengdu, 611130 Sichuan China; 2 Hangzhou Academy of Agricultural Sciences, Hangzhou, 310024 Zhejiang China

**Keywords:** Strawberry, Late embryogenesis abundant proteins, Fruit ripening

## Abstract

**Background:**

Late embryogenesis abundant (LEA) proteins play important roles in plant growth and development, as well as stresses responsiveness. Nowadays, it has been found that LEAs also have function in fruit ripening. However, the comprehensive analysis on a genome-wide basis of LEA family remains limited, and the role of *LEA* in fruit ripening has not been fully explored yet, especially in strawberry, an economic important plant and ideal material for studying fruit ripening.

**Results:**

In this study, a total of 266 putative LEA proteins were identified and characterized in strawberry genome. Subcellular localization prediction indicated that they were mostly localized in chloroplast, cytoplasm and nucleus. Duplication events detection revealed that whole genome duplication or segmental was the main driver for the expansion of LEA family in strawberry. The phylogenetic analysis suggested that FaLEAs were classified into eight groups, among which, LEA2 was the largest subgroup with 179 members, followed by LEA3, dehydrin (DHN), LEA4 and SMP (seed maturation protein). The LEA1 and DHN groups were speculated to play dominant roles in strawberry fruit development and ripening, according to their larger proportion of members detected as differentially expressed genes during such process. Notably, the expression of *FaLEA167* belonging to LEA1 group was altered by strawberry maturation, and inhibited by overexpression of negative regulators of ripening (a cytosolic/plastid glyceraldehyde-3-phosphate dehydrogenase, *FaGAPC2* and a cytosolic pyruvate kinase, *FaPKc2.2*). Subsequently, overexpression of *FaLEA167* significantly increased the percentage of fruit at green stage, while reduced the full red fruit proportion. In consistent, the anthocyanins content and the fruit skin color variable reflecting a range from greenness to redness (a* value) were significantly reduced. Whereas, *FaLEA167* overexpression apparently up-regulated citric acid, soluble protein and malondialdehyde content, but had no obvious effects on total soluble solids, sugar, flavonoids, phenolics content and antioxidant capacity.

**Conclusions:**

These findings not only provided basic information of *FaLEA* family for further functional research, but also revealed the involvement of *FaLEA167* in negatively regulating strawberry fruit ripening, giving new insights into understanding of *FaLEA* functions.

**Supplementary Information:**

The online version contains supplementary material available at 10.1186/s12864-024-10085-9.

## Background

Late embryogenesis abundant (LEA) proteins are a class of hydrophilic proteins with small molecular weight. They were initially isolated from embryos of mature wheat and cotton [1]. Moreover, LEAs extensively exist in plants, animals, as well as micro-organisms including bacteria and fungi [2, 3]. In plants, LEA proteins are commonly classified into eight groups namely LEA1, LEA2, LEA3, LEA4, LEA5, LEA6, dehydrin (DHN) and SMP (seed maturation protein) based on the sequence homology and conserved motifs available in the Pfam database [4, 5]. There are also some atypical LEA proteins lacking Pfam classification, which are classified into the AtM group that is specific to *Arabidopsis* and other *Cruciferae* [5]. Among all these groups, the common features are glutamic and glycine-rich and thermal stable proteins. In specific, LEA1 group proteins are highly conserved and characterized by the presence of an internal 20-amino acid signature motif [6]. By contrast, LEA2 group is different from other LEA groups and not conserved due to the less random coils and water stress and hypersensitive response (WHy) domain in secondary structure [7]. In addition, all DHN proteins contain K-segments, and some even have Y- or S-segments [8]. The apparent characteristic diversity for different LEA groups led to the hypothesis that these proteins may have multi-functional roles during plant development. To date, LEA proteins have been identified and analyzed at genome-wide level in various species, such as *Arabidopsis* [9], cucumber [10], lotus [11], wheat [12], orange [13] and potato [14]. However, the basic information including family members, evolutionary aspects, and functional fate of the LEA proteins in cultivated strawberry (*Fragaria* × *ananassa* Duch.) is still missing.

As the name implies, LEA proteins accumulate in the late stage of seed maturation, and play vital roles in seed formation and development. Besides, researches have indicated that LEA proteins also have functions in protecting plants from diversified abiotic stresses [15]. In fact, overexpression of *LEA* genes have improved the cold-, drought- and salt-tolerance of transgenic plants [16, 17]. Whereas, silencing of LEA4 group gene in *Arabidopsis* has resulted in water-deficit sensitivity [18]. The heterologous expression of wheat *LEA* also enhanced the tolerance of *E.coli* and yeast to salt and heat stress [19]. Moreover, it has been suggested that the expression of many LEA proteins is regulated by abscisic acid (ABA). For instance, the LEA4 members could be up-regulated by exogenous ABA and therefore involved in drought response during the development of maize embryos [20]. However, ABA is not only a key hormone in dehydration stress, but also one of the key regulators of strawberry fruit ripening [21]. The fact that fruit ripening has been recognized as an stress process, combined with their response to ABA might indicate the potential important roles of *LEA* genes in fruit ripening. Indeed, it has been previously suggested that the ABA-induced gene *CuLEA5*, one of the LEA5 group member, plays an important role in fruit ripening of *Citrus unshiu* [22]. The LEA members involved in strawberry fruit ripening need to be further identified.

Strawberry, a worldwide-cultivated economic fruit crop, is also an ideally and typical model plant for studying the non-climacteric fruit development and ripening. The excavation and utilization of related genes is an important way to regulate strawberry ripening to obtain high economic value and meet the supply–demand for cultivators and consumers. Our previous studies have implied that ABA and sucrose co-regulate strawberry fruit ripening positively [23]. while a cytosolic pyruvate kinase (*FaPKc2.2*) and cytosolic/plastid glyceraldehyde-3-phosphate dehydrogenase (*FaGAPC2*) were negative regulators of strawberry fruit ripening [24]. More important, we have found one gene, which belongs to the LEA family and was largely down-regulated by overexpression of *FaPKc2.2* and *FaGAPC2* genes in strawberry fruit. Therefore, we hypothesized that the *LEA* genes might play important roles in strawberry fruit ripening regulation. Based on the lack of basic information of *LEA* genes in strawberry, in this study, we have performed genome-wide identification and analysis of *LEA* genes by concentrating on the gene location, gene structure, evolutionary analysis and *cis*-elements analysis. The expression profiles of *LEA* genes during strawberry fruit development and ripening, in *FaGAPC2* and *FaPKc2.2* overexpression fruit samples were subsequently investigated. Moreover, the function of one *LEA* gene (*FaLEA167*) in strawberry ripening was further validated as well. The results here provided a better understanding of LEA family, and also gave insights into the putative roles of *LEA* genes in strawberry ripening.

## Materials and methods

### Identification and characteristic analysis of *FaLEA* genes

The Hidden Markov Model (HMM) file for each LEA group was downloaded from Pfam database (https://pfam.xfam.org) using ID PF03760, PF03168, PF03242, PF02987, PF00477, PF10714, PF00257 and PF04927. They were subsequently used as queries and searched against the protein sequence of cultivated strawberry, which was retrieved from the genome database for Rosaceae (https://www.rosaceae.org) [25]. The redundant and incomplete sequences were removed, and the remains were furtherly confirmed by searching conserved domain database [26]. A perl script was conducted to obtain the basic information of FaLEA proteins including deduced amino acids number, molecular weight (MW) and isoelectric point (pI). The locations of *FaLEAs* on chromosomes were retrieved from the genome annotation file, the conserved motifs were analyzed using MEME online program and visualized by TBtools software (v. 2.003). The subcellular localization prediction was performed by WOLF PSORT program (https://wolfpsort.hgc.jp).

### Phylogenetic, structure and *cis*-elements analysis of *FaLEAs* in strawberry

The multiple alignment of FaLEA proteins was carried out by online MUSCLE tools (https://www.ebi.ac.uk/Tools/msa/muscle/), a phylogenetic tree was then constructed using MEGA X software (v. 10.1.8) by maximum likehood method [27]. The tree was beautified by iTol (https://itol.embl.de/about.cgi) [28]. Duplication events and the collinear gene pairs were detected and visualized by MCScanX and Circos, respectively. Based on the genome annotation information, the exon–intron structures of *FaLEA* genes were obtained and visualized by TBtools (v. 2.003). The prediction of FaLEA proteins secondary structures were performed by online GOR IV method in the prabi database (https://npsa-prabi.ibcp.fr/cgi-bin/npsa_automat.pl?page=npsa_gor4.html), the alpha helix structures in FaLEA proteins were modeled in helical wheel diagram using HeliQuest database (https://heliquest.ipmc.cnrs.fr/). The average of α helix, β-sheet and random coil was calculated by using prabi online server and visualized using the part of whole function of Prism Graphpad (v. 9.0). The putative promoter regions were designated as 2 kb sequence upstream of the start codon in each *FaLEA* gene. Based on the genomic sequence and annotation information of strawberry, the promoter sequences of *FaLEA* genes were extracted and submitted to PlantCARE database (http://bioinformatics.psb.ugent.be/webtools/plantcare/html/) to identify the *cis*-elements.

### Plant materials and expression analysis

Strawberry ‘Benihoppe’ plants were grown in a greenhouse located in Chengdu, Sichuan Province, China, under the growth condition at of 24 ± 2℃, 75% relative humidity and a 14/10 h light/dark regime. The different plant tissues including root, stem, leaf, and flower were harvested from 5 plants and mixed as one replicate. Fruit at large green (LG), partial red (PR) and full red (FR) stages were collected on 15, 36, and 40 days after anthesis, respectively. Ten fruits were collected from 3 different plants and mixed as one replicate. A total of three replicates of each sample were constructed. The *FaGAPC2* and *FaPKc2.2* overexpression samples were collected in the previous study [24]. Fruit at white stage (W, at 22 days after anthesis) was used for transient transformation.

The transcriptome data-based expression of *FaLEAs* in different fruit developmental stages, in the *FaGAPC2*- and *FaPKc2.2*-overexpressed sample were estimated according to the previously published transcriptome data in NCBI sequence read archive database (accession: PRJNA838938), CNGB nucleotide sequence archive with accession number CNP0002459 and CNP0004133, respectively. The expression level was represented by FPKM values.

Quantitative PCR (qPCR) analysis were carried out using SYBR Green Premix Ex Taq™ (Takara, Japan) on a CFX96 qPCR system (Bio-Rad, USA). By using improved cetyltrimethylammonium bromide (CTAB) method and PrimeScript™ RT reagent Kit (Takara, Japan), total RNA was isolated and the first strand cDNA was synthesized. The relative expression was calculated using the 2^−ΔΔCt^ method [29]. The 26-18S interspacer RNA sequence was employed as the internal reference. Expression data was represented by mean ± standard deviation (SD) of three independent biological replicates. All the primer sequences used in this study were listed in Supplementary Table 6 (Table S6).

### Transient overexpression of *FaLEA167* gene

The full length of *FaLEA167* CDS was amplified and substantially inserted into a modified overexpression (OE) vector [30]. The agrobacterium containing recombinant plasmid was cultured to OD600 0.8 ~ 1.0, then 500 μL of the suspension was injected into each fruit [30]. After 7 days of cultivation in an incubator under the condition of 24 ± 2℃, 75% humidity, the injected part of fruit was sampled and stored at -80℃ for further use. The fruit injected with empty vector was used as control. At least 30 fruits were injected for OE and control group separately.

### Determination of fruit skin color, firmness, total soluble solids, and soluble sugar

The skin color of the strawberry was recorded by a CR-400 chromometer (Konica Minolta, Tokyo, Japan), and represented by L*, a*, and b* variables. The L* value indicates darkness to lightness; the a* value suggests a range from greenness to redness; and the b* value indicates blueness to yellowness.

Fruit firmness represented as newton (N) was measured via a Texture Analyzer TA XT2i (Stable Micro systems, Godalming, Surrey, UK) with a 5 mm diameter cylinder needle. A digital pocket refractometer (PAL-1, Atago, Tokyo, Japan) was used for total soluble solids (TSS) determination. Soluble sugar content was detected by the colorimetric method. The absorbance of the extraction solution was read at 620 nm, and an external standard was employed to quantify the soluble sugar content.

### Malondialdehyde and soluble proteins

The malondialdehyde (MDA) was determined using the previously reported method [31]. Briefly, a complete homogenization of approximate 0.5 g of fruit was achieved by 10% trichloroacetic acid, the mixture was then centrifuged for 10 min at 4 ℃, the clear solution was extracted and mixed with 0.67% 2-thiobarbituric acid. After a 10 min water bath at 100 ℃, it was immediately cooled on ice. The absorption value at 450 nm, 523 nm, and 600 nm was separately recorded. The MDA content was represented as μmol per g fresh weight (FW). The soluble protein content was assayed according to the formerly demonstrated procedure with slight modifications. In a brief, 5 ml of distilled water was used to homogenize 0.5 g of fruit sample. CBBG was added to the upper phase, and subjected to centrifugation. The photographic density of 595 nm was tested. Bovine serum albumin (BSA) protein was used to construct a standard curve for the soluble protein was quantification.

### Total flavonoids, phenolics, and anthocyanins detection

According to the previously described procedures [32, 33]. 5 mL of 80% acetone was used for extraction of about 3 g of fruit. After 1 h reaction at room temperature, the extraction was centrifuged for 10 min at 4500 rpm, the supernatant was then collected for total flavonoids and phenolic content detection. The absorbance of the mixture at 415 nm and 650 nm was recorded, and the quercetin and gallic acid were used as external standards for total flavonoids and phenolic content calculation, respectively. The total flavonoids content was presented as mg quercetin per kg of FW, and the total phenolic content was expressed as g gallic acid per kg of FW.

The determination of total anthocyanins was performed by pH differential method [34]. As previously described, acetic acid: water: acetone: methanol (1:2:4:4) solution was used for extraction. The fruit was extracted for 30 min at room temperature, and then incubated at 40 ℃ for 4 h. The clear extract was added with KCl (0.025 M, pH 1.0) and sodium acetate, and then detected by recording the absorption value of 496 and 700 nm. The content of total anthocyanins was expressed as g pelargonidin 3-glucoside per kg of FW.

### Total antioxidant activity

Total antioxidant activities were estimated by the determination of FRAP (ferric-reducing antioxidant power) and DPPH (2,2-diphenyl-1-picrylhydrazyl). The FRAP was measured according to the previous approach [35]. The working FRAP reagent was made freshly, and was consisted of 300 mM acetate buffer (pH 3.6), 10 mM TPTZ, and 20 mM FeCl_3_·6H_2_O in the ratio of 10:1:1 (*v*/*v*/*v*). The mixture of sample extract and reagent was allowed to react for 30 min, the absorbance at 593 nm was recorded to estimate FRAP and the result was denoted as mmol kg^−1^ FW. For DPPH assays, previous method was used [36]. methanol was used to extract 0.1 g of sample for 2 h. After then, 500 μL supernatant was collected by centrifuge, and added with DPPH solution in methanol. After a 30 min reaction in dark at room temperature, the absorption value at 517 nm was detected.

## Results

### Identification of *LEA* family genes in strawberry

A total of 266 *FaLEA* genes were identified in cultivated strawberry genome (Table S1). According to their chromosomal locations, all the 266 *FaLEA* genes were designated as *FaLEA1* to *FaLEA266* (Fig. S1). As shown, 266 *FaLEA* genes were unevenly distributed across the 28 chromosomes in the 4 subgenomes of cultivated strawberry, with an obvious concentration on the chromosomes 5, 6 and 7. A maximum of 27 and a minimum of 2 *FaLEA* genes were located on chromosome 5 and 3 from the fourth subgenome (Fvb5-4), respectively (Fig. S1). According to the subcellular localization prediction results, most FaLEA proteins were predicted to be located in the chloroplast (115), cytoplasmic (55) and nuclear (50), some FaLEA proteins were localized in mitochondria, endoplasmic reticulum, plasma membrane, vacuole, peroxisome and extracellular matrix. Interestingly, a few FaLEA proteins were predicted to be dual-localized, as examples, FaLEA265 was located in both cytoplasm and nucleus, while FaLEA140 was located in endoplasmic reticulum or plasma membrane (Table S1). Furthermore, the origins of duplication events of *FaLEA* genes in strawberry were tested using MCScanX package. As a result, 232 and 23 *FaLEA* genes were duplicated from whole genome duplication or segmental, and dispersed separately. *FaLEA182/50/51* were involved in proximal duplication event, *FaLEA166/240/54/53/52* were detected as tandem duplicated, while *FaLEA85* was singleton duplicated (Table S1).

### Classification and characteristics of FaLEA proteins

On the basis of different Pfam domain types, all the 266 FaLEA proteins were classified into 8 subgroups: LEA1, LEA2, LEA3, LEA4, LEA5, LEA6, DHN and SMP. Among these, LEA2 was the largest subgroup with 179 members, followed by LEA3, DHN, LEA4 and SMP, which contained 19, 18, 14 and 12 members respectively. The LEA5 subgroup was consisted of 11 members, while the LEA1 subgroup included 8 members. The LEA6 was the smallest subgroup with only 5 *FaLEA* members (Table S1).

**Fig. 1:**
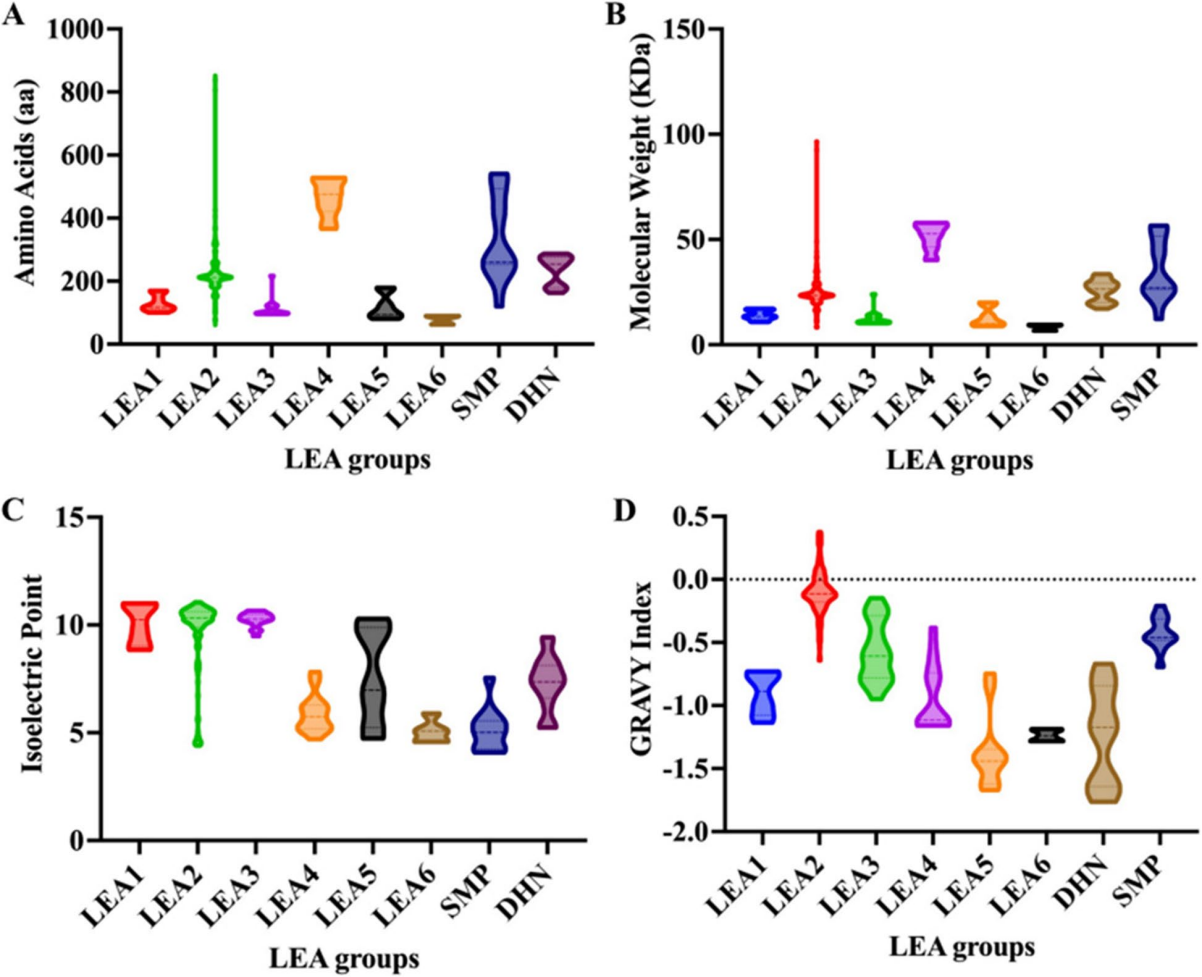
Physicochemical properties of the FaLEA proteins. **A** Amino acids length of FaLEA proteins. **B** Molecular weight of FaLEA proteins. **C** Isoelectric point and (**D**) GRAVY index of FaLEA proteins

The characteristics and physicochemical properties of the deduced 266 FaLEA proteins were estimated (Fig. 1, Table S1). Although the number of amino acids varied from 62 to 837 aa, 78% of them (207 out of 266) were concentrated from 100 to 300 aa. LEA2 subgroup had the broadest range, but only two members had an amino acids length greater than 800 aa. Group LEA4 had the largest length in amino acids with all members greater than 350 aa, while group LEA6 had the smallest amino acids length, with all members less than 100 aa (Fig. 1A). The MW were from 6.686 to 96.497 KDa. Similar to amino acids length, the broadest range of MW was found in LEA2 subgroup. All members belonging to LEA4 subgroup had MW greater than 40 KDa, while the MW of members in LEA6 group was less than 10 KDa (Fig. 1B). Only 56 FaLEA proteins had pI below 7, while the others were all above 7 and 153 of which contained pI above 10. Group LEA1 and LEA3 had pI larger than 7 and group LEA6 had pI smaller than 7 (Fig. 1C). For grand average of hydropathicity (GRAVY) index, 37 FaLEA proteins from group LEA2 with indices greater than 0 were considered as hydrophobic, while the remaining were hydrophilic proteins with indices less than 0 (Fig. 1D).

### Phylogenetic, gene structure and motif analysis

**Fig. 2:**
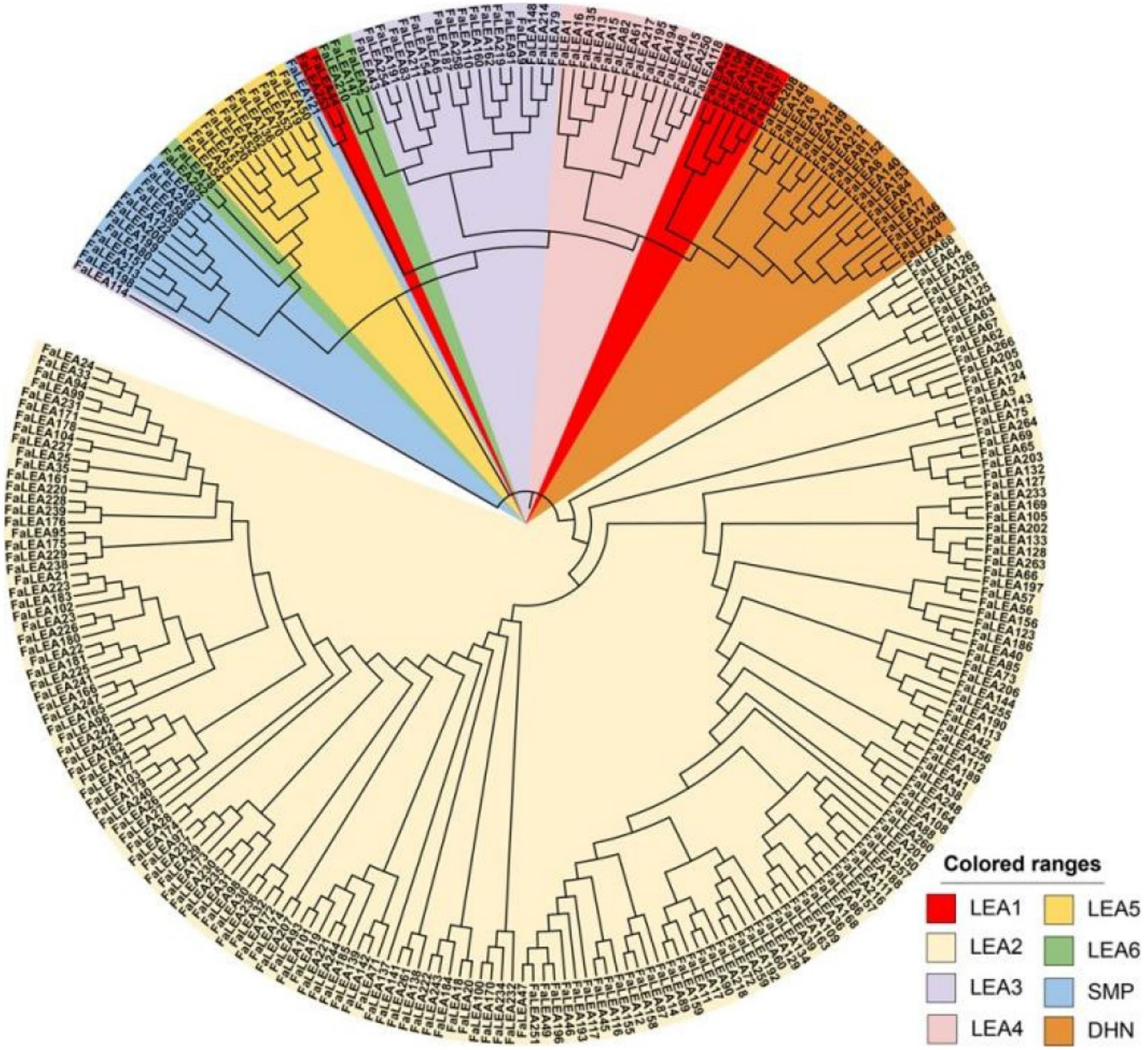
Phylogenetic analysis of FaLEA proteins in strawberry. Different colors indicated different subgroups of FaLEAs

A phylogenetic tree was constructed based on multiple proteins alignment (Fig. 2). It was suggested that most LEA proteins within the same subgroup could cluster into a single clade, except for those in LEA1, LEA3, LEA6 and SMP subgroups, which could be divided into two sub-clades (Fig. 2).

In addition, the intron/exon distributions of *FaLEA* genes were analyzed and visualized (Fig. S2). Overall, the exon numbers in the *FaLEA* genes ranged from 1 to 7. Among all the 8 subgroups, most groups contained 2 to 3 exons, the LEA1 group concluded 2 exons, while the LEA6 group only had one exon. It was noted that three *FaLEAs* members (*FaLEA58, FaLEA59 and FaLEA122*) in group SMP contained the most exons, while the other nine members of *FaLEAs* had a range from 2 to 6 of exons.

**Fig. 3:**
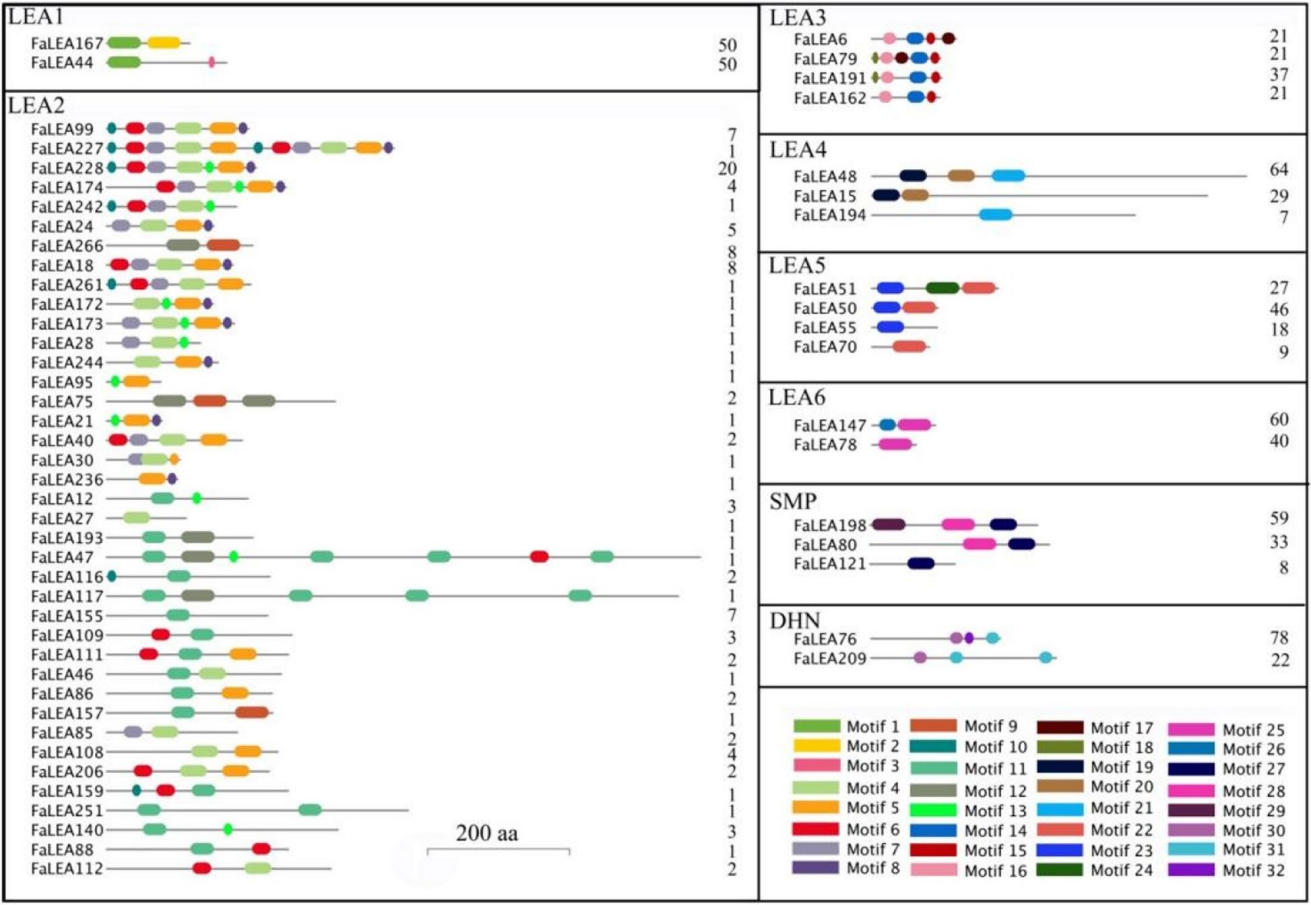
Representative motifs distribution in FaLEA protein family. The numbers at the end of each protein sequence presented the percentage of FaLEAs with the same motif pattern. The bar represented 200 aa

Meanwhile, the conserved motifs analysis of FaLEA proteins suggested that the motifs number and distribution order were similar within the same FaLEA group, and each group contained the group-specific conserved Pfam domains (Fig. S3). The representative distribution patterns of motifs were displayed in Fig. 3. It was showed that, LEA1, LEA6 and DHN group members involved in 2 distribution patterns respectively. LEA3 and LEA5 group members contained 4, while LEA4 and SMP group members had 3 types of distribution patterns. 38 patterns of motif distribution exhibited in LEA2 members (Fig. 3).

### Collinearity analysis of *FaLEA* genes

**Fig. 4:**
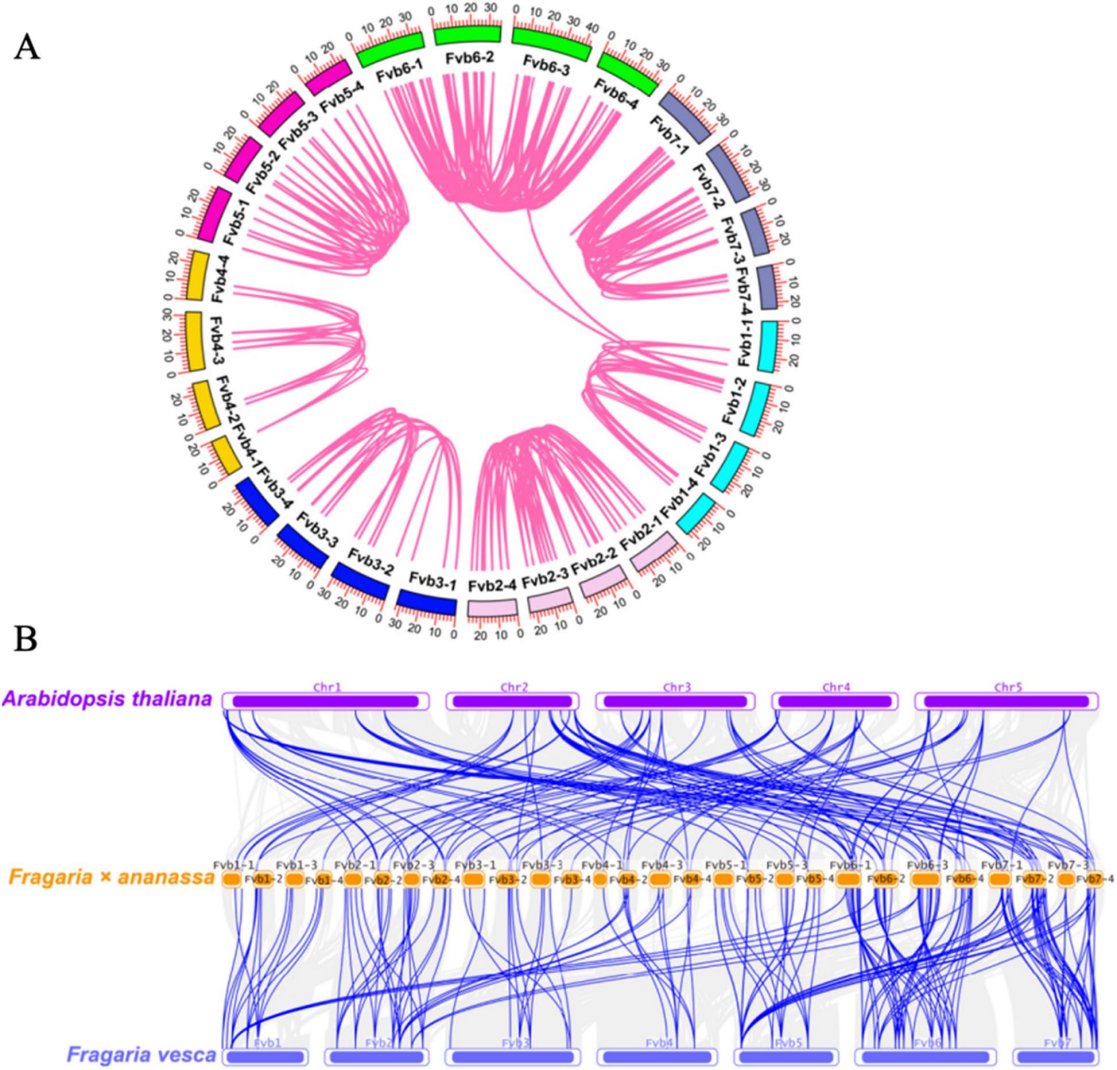
Synteny and collinearity analysis of LEA genes. **A** synteny analysis of FaLEA genes in cultivated strawberry. Chromosomes were discriminated by different colors. Pink curves indicated collinear pairs. **B** Collinearity analysis of LEA genes among *Arabidopsis thaliana*, *Fragaria* × *ananassa* and *Fragaria vesca* genomes. Grey lines indicated collinear blocks within the three genomes, while the blue lines represented collinear LEA gene pairs. The purple, yellow and blue columns indicated the chromosomes from *Arabidopsis thaliana*, *Fragaria* × *ananassa* and *Fragaria vesca* genomes respectively. Chromosomes number were displayed at the side of chromosomes

To clarify the evolutionary relationship of *LEA* genes, the collinearity analysis within strawberry genome, among *Arabidopsis*, woodland strawberry (*Fragaria vesca*) and cultivated strawberry (*Fragaria* × *ananassa*) genome was performed. According to the result, there were 284 collinear pairs of *FaLEA* genes in cultivated strawberry (Table S2 and Fig. 4A). All of the *FaLEA* pairs co-located in the same or adjacent chromosome, except for *FaLEA73* (chromosome Fvb1-2), which was collinear with *FaLEA190* (chromosome Fvb6-3) and *FaLEA42* (chromosome Fvb6-1). Additionally, multiple genome comparison revealed that 42 *AtLEA*, 46 *FvLEA* and 179 *FaLEA* genes were involved to form a total of 348 collinear pairs (Table S2). In specific, 120 pairs identified between *Arabidopsis* and cultivated strawberry, and 228 pairs found between woodland strawberry and cultivated strawberry were highlighted in Fig. 4B.

Furthermore, the calculation of number of non-synonymous substitutions per non-synonymous sites (Ka), the number of synonymous substitutions per synonymoussites (Ks) and the Ka/Ks ratio for paralogous gene pairs of *FaLEA* was carried out (Table S3). According to the results, the Ka, Ks and Ka/Ks ratio varied from 0 to 1.16, 0 to 1.84, and 0 to 4.35, respectively. The majority of *FaLEA* pairs had a Ka/Ks ratio less than one, while 21 pairs had a Ka/Ks ratio greater than one (Table S3), suggesting they were under positive selection during the evolution process.

### Secondary structural characteristics of FaLEA proteins

**Fig. 5:**
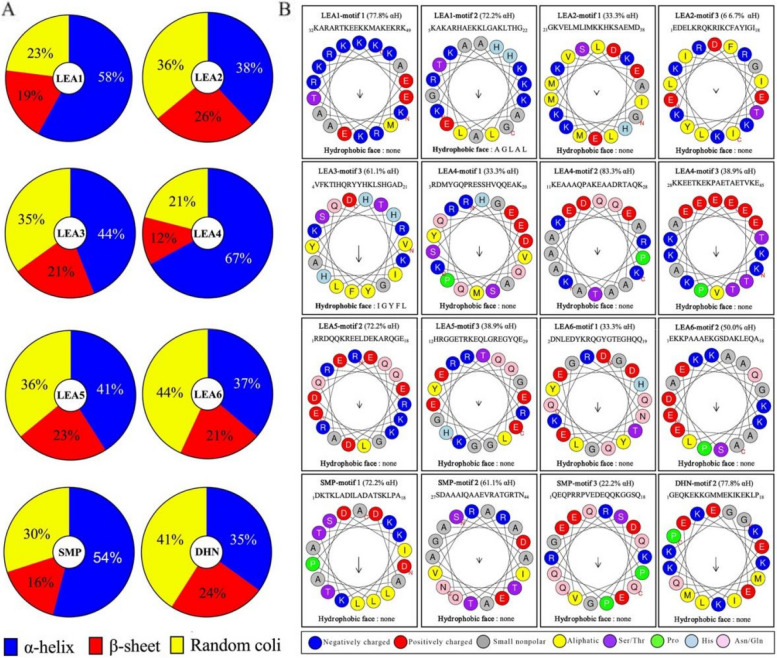
secondary structure prediction of FaLEA proteins. **A** average distribution of α helix, β-sheet and random coil of FaLEA proteins in each group. **B** Modelling of α helix in FaLEA proteins. The conserved motifs detected by MEME were submitted to generate α helix structure by using HeliQuest webserver. αH indicated the percentages of α helix structure. Each wheel was obtained with 1_TURN window size. The arrow suggested the helical hydrophobic moment

Structure analysis revealed that FaLEA proteins in distinct groups differed in their secondary structure. Overall, the proportion of α helix, β-sheet and random coil ranged from 35 to 67%, 12 to 26% and 21 to 44%, respectively. Particularly, the LEA1, LEA4 and SMP group members had high propensity to form α helix, with a distribution of 58%, 67% and 54%. Whereas, random coil was found to be the prevalent protein conformation in LEA6 and DHN members, and each had a percentage of 44 and 41. Moreover, LEA2, LEA3 and LEA5 group members had similar distribution of α helix, β-sheet and random coil (Fig. 5A).

To obtain a better understanding of the α helix occurrence, the structural properties of conserved motifs in each FaLEA group were analyzed. As a consequence, motif 1 and 2 in the LEA1 group displayed high possibility to form negative charged α helix structure, with or without hydrophobic faces, separately (Fig. 5B). Motif 1 and motif 3 in LEA2 group exhibited a tendency to form α helix structure, but no hydrophobic face was found. On the contrary, the motif 3 was predicted to form α helix with hydrophobic face. In the LEA4 group, distinct charged α helixes without hydrophobic face were predicted in motifs 1, 2 and 3. Motif 2 and 3 in LEA5, motif 1 and 2 in LEA6 group, motif 1, 2 and 3 in SMP group intended to form α helixes with no hydrophobic faces. Only motif 2 was predicted to form α helix in DHN group (Fig. 5B).

### *Cis*-element analysis of *FaLEA* genes

**Fig. 6:**
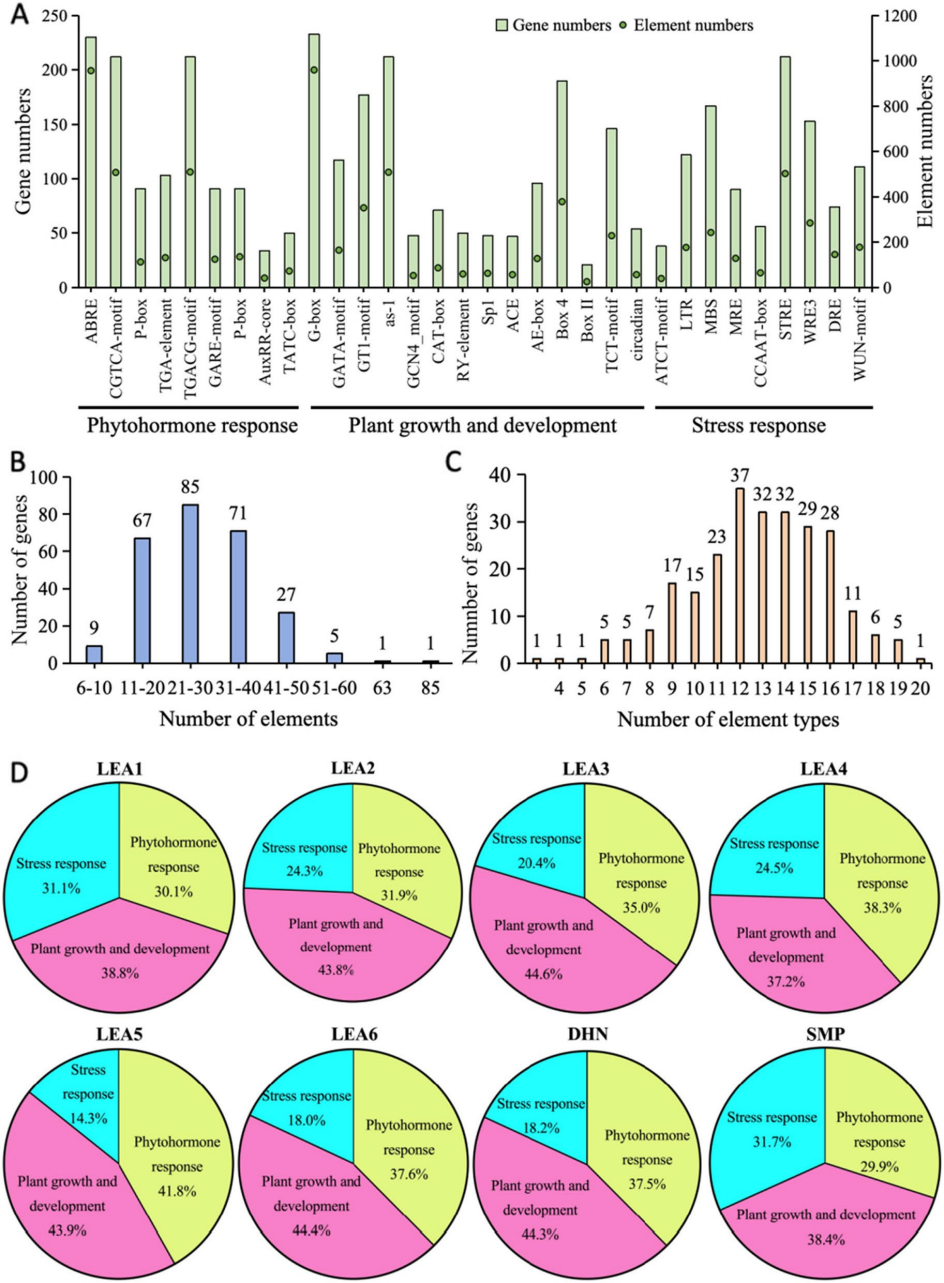
Analysis of the *cis*-elements in the putative promoter regions of *FaLEA* genes. **A** The numbers of genes and corresponding elements in their promoters. Three major types of elements were classified based on elements functional annotation. **B** Elements number distribution in *FaLEA* genes. **C** Distribution of types number in *FaLEA* genes. **D** Distribution of elements types among each FaLEA group

The analysis of *cis*-elements in the putative promoter regions of *FaLEA* genes was conducted. It was suggested that, 266 *FaLEA* genes contained a total of 7487 *cis*-elements falling into 32 types (Table S4). Among them, the most *cis-*elements were associated with plant growth and development, followed by phytohormone response, and stresses response (Fig. 6A). Specifically, various *FaLEA* genes contained a large number of motifs that were involved in light response, such as G-box (960), as-1 (509), Box 4 (379), GT1-motif (352) and TCT-motif (229). Besides, several other regulatory cis-elements that were involved in light responsive (GATA-motif and AE-box), endosperm expression (GCN4_motif), seed-specific regulation (RY-element), as well as circadian control (circadian) were found in the *FaLEA* promoters during plant growth and development. Among the phytohormones responsive elements, methyl jasmonate response elements (CGTCA-motif and TGA-motif) were the most (1018), followed by ABA responsive elements (ABRE) with a number of 952, gibberellin-responsive elements (125 GARE, 136 P-box, and 73 TATC-box), and auxin responsive elements (132 TGA-element and 42 AuxRR-core). Moreover, stress response elements (STRE), wounding and pathogen responsiveness (WRE3 and WUN-motif), and MYB binding site involved in drought-inducibility (MBS) comprised of the most elements related to stress responsive. Furthermore, most *FaLEA* genes contained 21–30 *cis*-elements (Fig. 6B), among which, *FaLEA85* contained the largest number of elements (85), while *FaLEA72* had the smallest elements number (6). The types of elements included in each *FaLEA* genes varied (Fig. 6C), with an apparent concentration of 12 to 14. Among the distinct LEA groups, it was showed that majority of LEA groups contained the most elements related to plant growth and development, while the least elements associated with stress responsiveness, except for LEA1, LEA4 and SMP groups (Fig. 6D). The LEA1 and SMP groups had similar distribution of elements, with a maximum of that involved in plant growth and development (around 38%) and minimum of that responsive to phytohormones. However, the LEA4 group contained most of elements that were interrelated to phytohormone responsiveness, followed by plant growth and development, and stress responsiveness.

### Identification of *FaLEA* genes involved in strawberry development and ripening

**Fig. 7:**
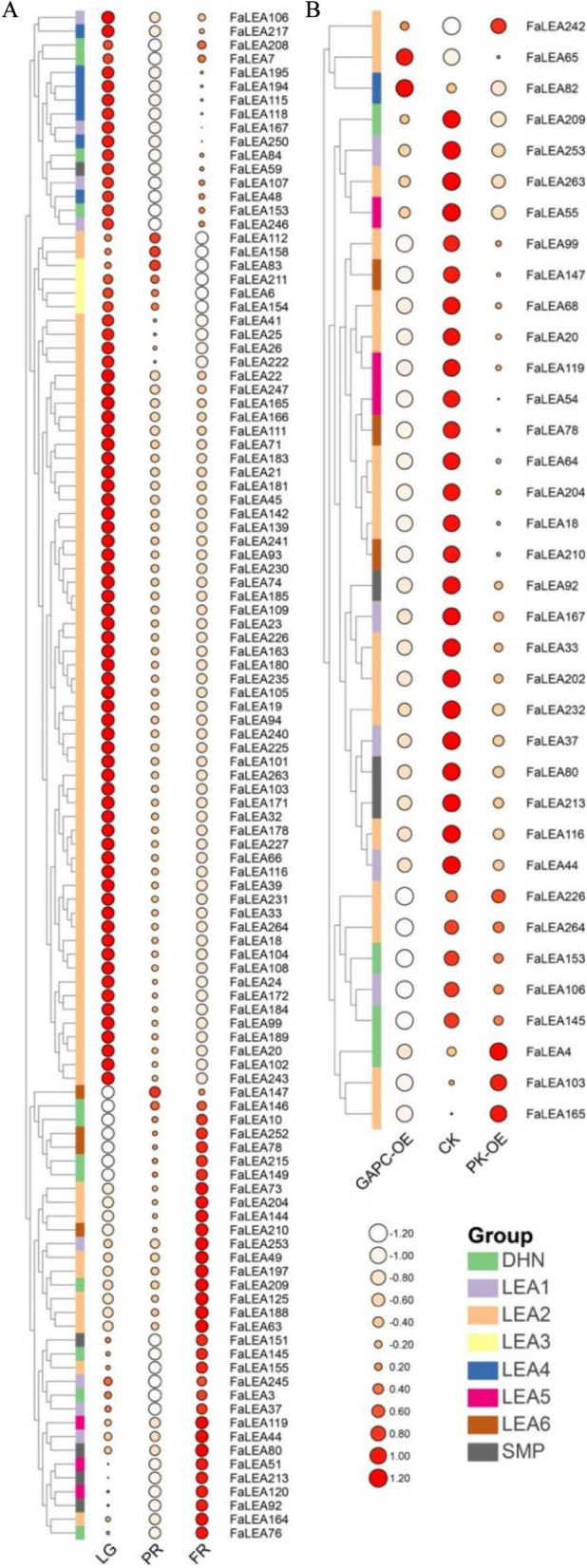
RNAseq based expression profiles of *FaLEA* genes. **A** Heatmap showing the expression of *FaLEA* genes during fruit development and ripening. **B** Transcript abundance of *FaLEA* genes in *FaGAPC2*- and *FaPKc2.2*-overexpressed fruit. The expression levels were presented by FPKM value. Heatmaps were normalized by rows. LG, large green; PR, partial red; FR, full red. OE, overexpression

To explore their prospective roles in the development and ripening of strawberry fruit, the expression patterns of 266 *FaLEA* genes were estimated based on previously published transcriptome data (Table S5). As displayed in Fig. 7A, 111 *FaLEA* genes were differentially expressed during the fruit development. Among them, all the LEA1 group members were included, while only around 39%, 21%, 57%, 27%, 80%, 33% and 56% of LEA2, LEA3, LEA4, LEA5, LEA6, SMP and DHN members were included, respectively. Generally, most *FaLEA* genes showed high expression levels in large green (LG) stage, while exhibited low expression in full red (FR) stage. Whereas, several *FaLEA* genes showed completely contrary expression patterns with higher expression levels in FR (Fig. 7A). Particularly, all of the included LEA5, and most of the SMP members showed the lowest expression levels at PR stage, and the highest levels at FR stage. A large proportion of LEA4 members displayed the highest expression at LG stage, while the lowest expression at PR stage. All the LEA6 members exhibited gradual increase expression trend, while all the LEA3 members and most of the LEA2 members conferred gradual decline in expression during development and ripening (Fig. 7A). This result revealed the potentially different roles of *FaLEA* genes in strawberry ripening.

Furthermore, 36 *FaLEA* genes showed different expression levels in *FaGAPC2* and *FaPKc2.2* OE samples comparing to the control (Fig. 7B). Among them, 30 *FaLEA* genes were inhibited in expression both by OE of *FaGAPC2* and *FaPKc2.2* genes. However, an opposite change in expression under *FaGAPC2* and *FaPKc2.2* OE was observed for the genes *FaLEA242* and *FaLEA65*. *FaLEA82* was induced by *FaGAPC2* OE but repressed by *FaPKc2.2* OE. By contrast, an inhibition in *FaGAPC2* OE sample while an upregulation in *FaPKc2.2* OE sample were found in *FaLEA4*, *FaLEA103* and *FaLEA165* expression (Fig. 7B).

**Fig. 8:**
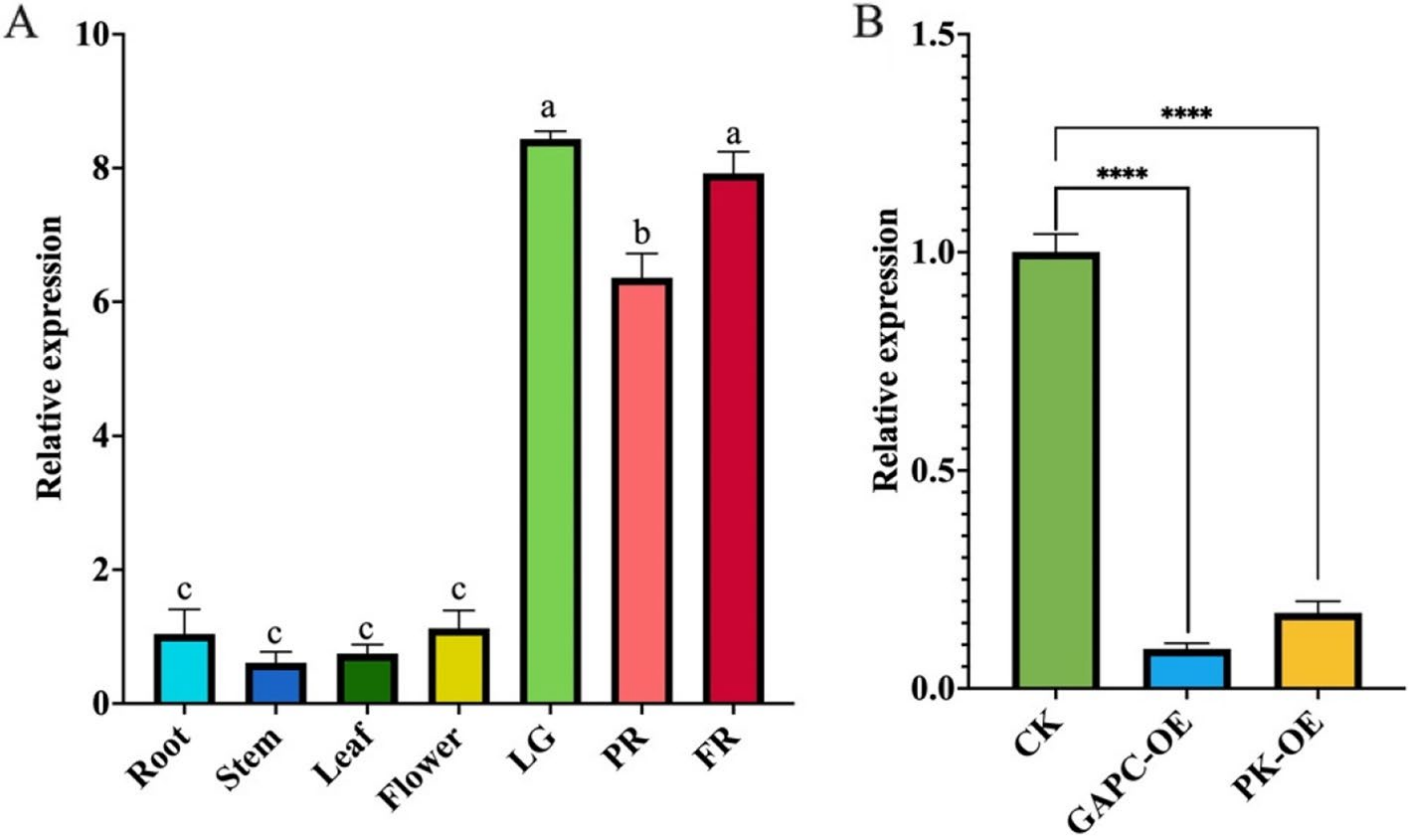
Expression analysis of *FaLEA167* gene by qPCR. **A** The relative expression of *FaLEA167* in different fruit tissues and during fruit development and ripening. Lowercase letters suggested the statistical significant difference at *P* ≤ 0.05 level. **B** Transcript level of *FaLEA167* in the *FaGAPC2*- and *FaPKc2.2*-overexpressed fruit. Asterisks indicated significant statistical difference at *P* ≤ 0.01 level. LG, large green; PR, partial red; FR, full red. OE, overexpression

Notably, a gene namely *FaLEA167* was found differentially expressed during fruit development, as well as in *FaGAPC2* and *FaPKc2.2* OE samples, indicating that it was possibly related to strawberry ripening. Therefore, *FaLEA167* was selected for subsequent qPCR expression analysis. As a result, *FaLEA167* exhibited higher expression level in fruit than other tested tissues including root, stem, leaf and flower (Fig. 8A). Particularly, during fruit development and ripening, the expression of *FaLEA167* was detected at the highest level in LG stage, a subsequent decline and then increase was observed in PR and FR stage, respectively. Although a lower *FaLEA167* expression level was observed in FR compared to LG stage, no statistical significant difference was found (Fig. 8A). In addition, the expression of *FaLEA167* was significantly reduced by *FaGAPC2* and *FaPKc2.2* OE (Fig. 8B), which was in consistent with the transcriptome data.

### Overexpression of *FaLEA167* in strawberry fruit

**Fig. 9:**
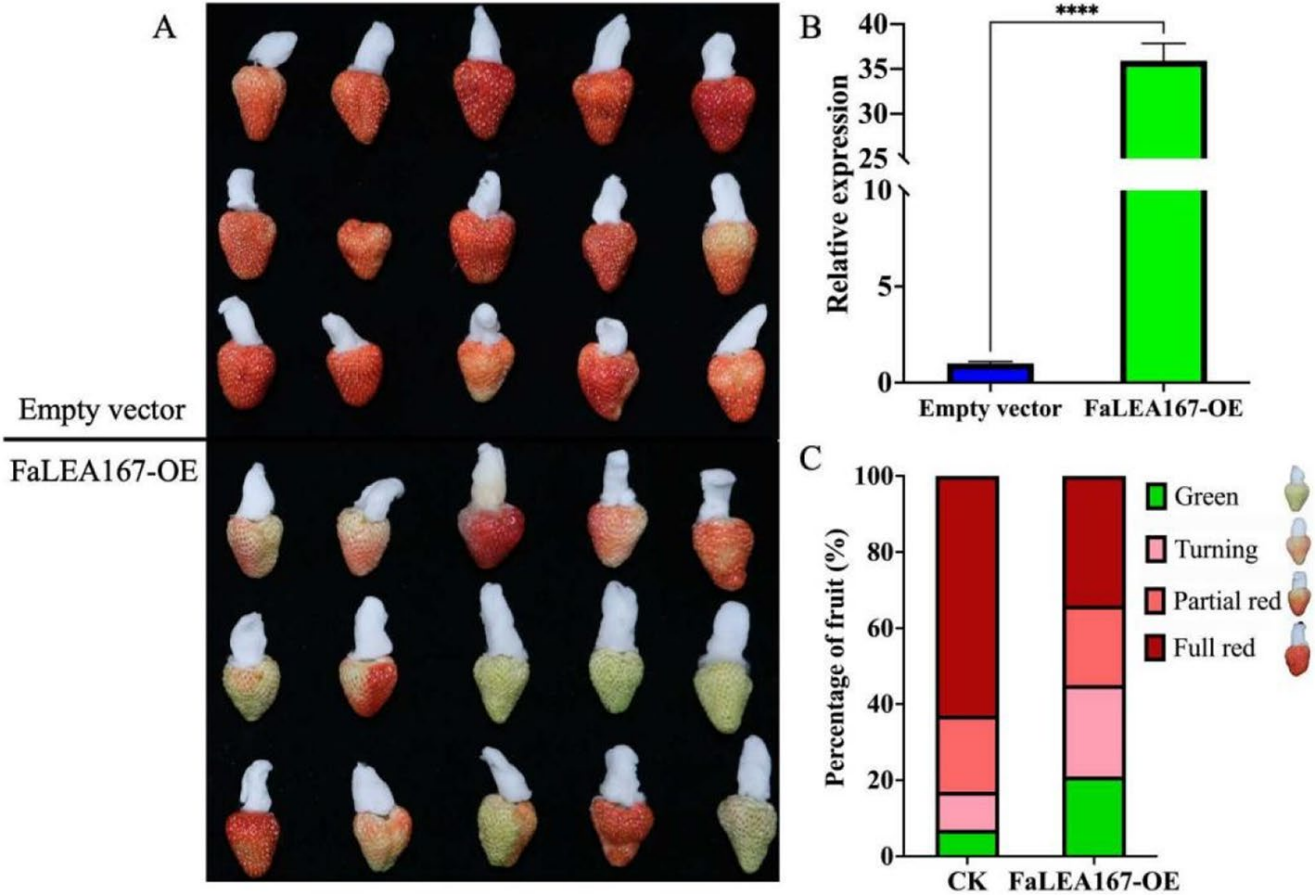
Overexpression of *FaLEA167* in strawberry fruit. **A** The phenotype of strawberries injected with empty vector (control) and *FaLEA167*-overexpressing recombinant plasmid. **B** The relative expression of *FaLEA167* in overexpressed and control fruit. **C** The percentage of fruit at different developmental and ripening stage in control and *FaLEA167*-overexpressed fruit. OE, overexpressing. Asterisks represented statistical difference at *P* ≤ 0.001 level

Subsequently, *FaLEA167* was transiently overexpressed in strawberry fruit to validate its function in ripening. The phenotypical result showed that, OE of *FaLEA167* obviously and significantly repress the fruit coloring (Fig. 9A). Around 30 times higher expression level of *FaLEA167* was found in the overexpressed sample compared to the control (Fig. 9B), indicating that *FaLEA167* was successfully overexpressed. Moreover, the percentage of FR fruit was significantly decreased, while the percentages of PR and green fruit were largely increased by OE of *FaLEA167* (Fig. 9C), confirming the potential negative regulatory role of *FaLEA167* in strawberry fruit ripening.

### The effects of *FaLEA167* overexpression on fruit ripening-related traits

**Fig. 10:**
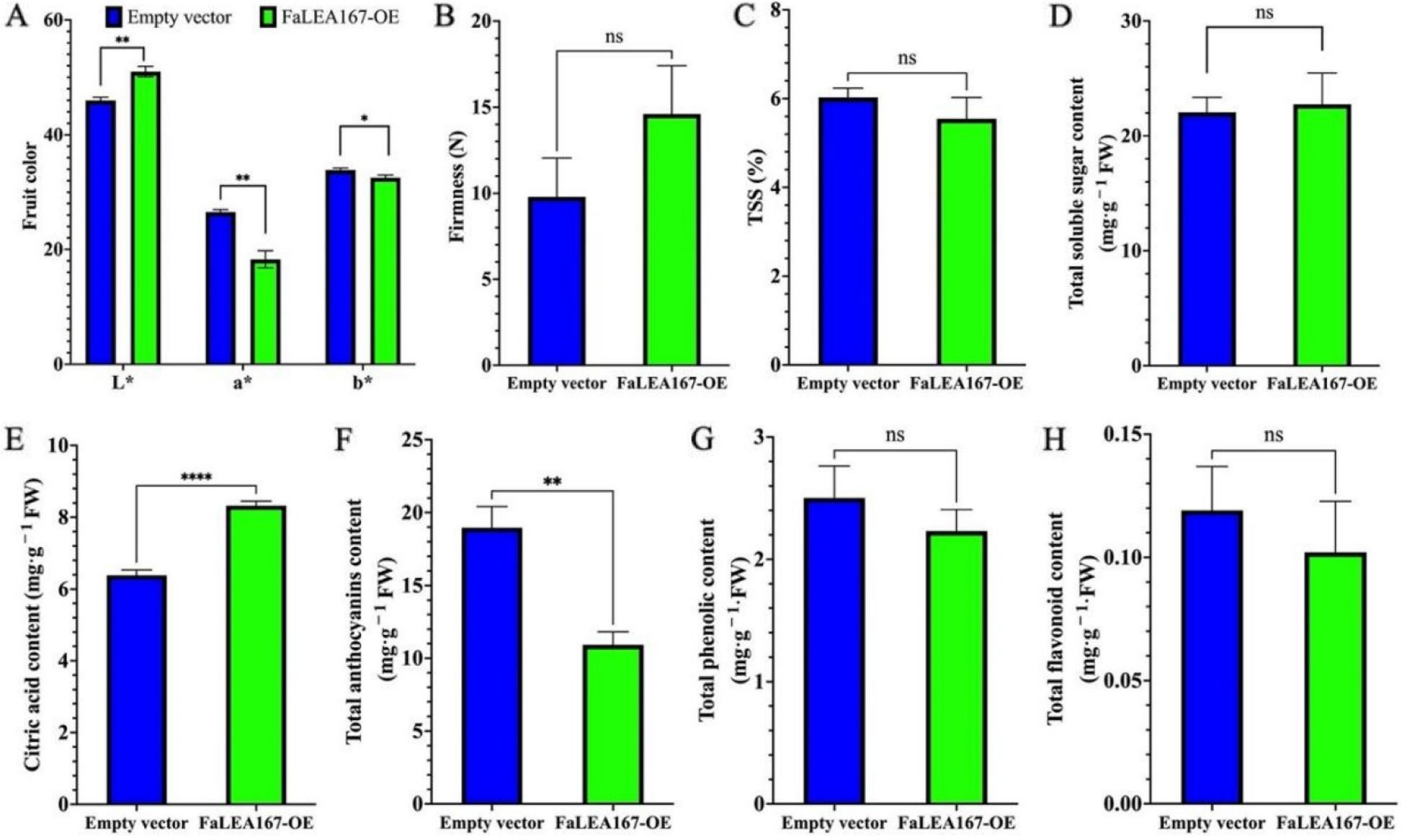
The effects of *FaLEA167* overexpressing on the ripening-related traits. **A**-**H** indicated fruit color, fruit firmness, total soluble solids content, total soluble sugar content, citric acid content, total anthocyanins content, total phenolic and flavonoid content, respectively. Asterisks indicated statistical difference at *P* ≤ 0.01 level. Ns, no significant statistical difference was found

There were significant differences in L*, a*, and b* between the empty control group and the *FaLEA167* OE group. Among them, the a* and b* of *FaLEA167* OE fruit were significantly lower than the empty control group, while L* was significantly higher than the empty control group (Fig. 10A), indicating that OE of *FaLEA167* can delay fruit coloring but also increase fruit brightness. In addition, it was found that there was no significant difference in fruit firmness (Fig. 10B), TSS (Fig. 10C), total sugar (Fig. 10D), total phenolic and total flavonoids content (Fig. 10G and Fig. 10H) between OE and control fruit. Notably, the citric acid and anthocyanin content were significantly increased and decreased in *FaLEA167*-overexpressed fruit (Fig. 10E and Fig. 10F), respectively, suggesting that OE of *FaLEA167* can delay fruit ripening.

**Fig. 11:**
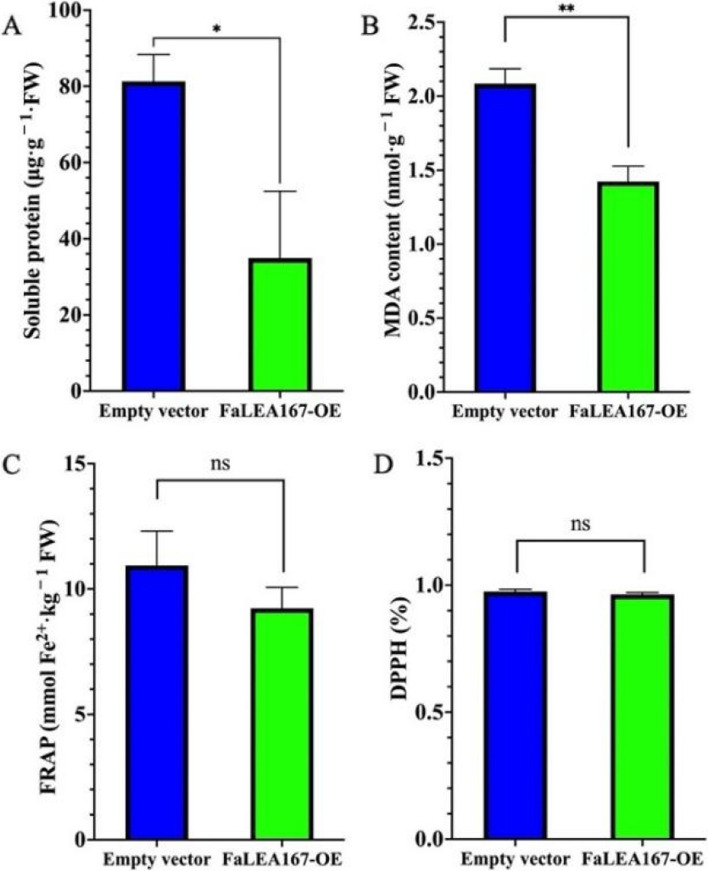
Changes of antioxidant-related traits in control and *FaLEA167* overexpressed fruit. **A** The content of soluble protein. **B** MDA content. **C** and (**D**) indicated the FRAP and DPPH, respectively

Furthermore, it was found that there were significant differences in the content of soluble protein and MDA in *FaLEA* OE group (Figs. 11A and B), which was significantly lower than that in the control group. However, there was no significant difference in FRAP and DPPH (Fig. 11C and Fig. 11D), indicating that the OE fruit conferred less oxidative stress.

## Discussion

*LEA* genes have critical functions in embryonic development and stress response. They are distinctly distributed in various species, with a small number of 27, 28, and 33 in tomato [37], lotus [11] and tea plant [38], but a comparative larger number of 43, 181 and 318 in grape, apple [39] and *Ramonda serbica* [40], respectively. The larger number in the latter species might be caused by an evolutionary advantage of LEA proteins in the drought tolerant species, since LEA proteins have been recognized as a hallmark of desiccation tolerance [40–42]. However, strawberry is a dehydration-sensitive plant, and a sum of 266 *FaLEA* genes were identified (Table S1). This is probably due to the whole genome duplication of cultivated strawberry during the evolutionary process [43]. The expansion and evolution of gene families are driven by gene duplication events, among which, segmental and tandem have been suggested as two main types of duplication [44, 45]. In support of this, segmental was found as the predominant driver of lotus LEA gene family expansion in the previous study [11] and our results (Table S1). Moreover, although LEA proteins can be divided into eight groups in several plants, the number of each group varies. It has been previously suggested that the LEA4 group was the most dominant in *Arabidopsis*, lotus and grape genome [5]. Whereas, LEA2 group was found to be the most abundant in tea plant, cotton and across 60 complete plant genome, encompassing around 40% and 65% of all LEA proteins separately [38, 46]. Similarly, our results showed that LEA2 group contained the most members, encompassing about 67% of all FaLEA proteins (Table S1). A possible reason for the changes of group member number might be the differentiation of expansion rapid in each group.

The identification of various *cis*-elements in the promoter regions of *FaLEA* genes revealed multiple functions of *FaLEA* in phytohormone response, plant growth and development, and stress response (Fig. 6 and Table S4). Indeed, besides of their well-known roles in development and stress response [14, 40], seed development [11], protection of proteins from folding of denatured proteins [47], and resisting cellular damage [48]. LEA proteins have been suggested to be involved in fruit ripening regulation [22]. To identify the *LEA* members related to strawberry ripening, the expression profiles of *FaLEA* genes during fruit development and ripening were investigated (Fig. 7). Among the 111 differentially expressed *FaLEA* genes, the LEA1 and DHN groups were found to encompass the widest range of expression patterns (Fig. 7). These differences in expression patterns of each group might be caused by the group-specific function as previously suggested [11], and the LEA1 and DHN groups were speculated to play more important roles in strawberry fruit development and ripening. Moreover, about 63% (5 out of 8) of LEA1 members, but only around 30% of DHN members were differentially expressed in the OE of *FaGAPC2* and *FaPKc2.2* fruit. Therefore, one of the LEA1 member *FaLEA167* was selected for further functional analysis. Notably, *FaGAPC2* and *FaPKc2.2* regulated strawberry fruit ripening negatively and repressed *FaLEA167* expression, suggesting that *FaLEA167* should be a positive regulator of strawberry fruit ripening. However, our subsequent functional analysis showed that *FaLEA167* OE inhibited strawberry fruit ripening (Fig. 9). This contradiction may be explained by the fact that *FaGAPC2* and *FaPKc2.2* inhibited strawberry fruit ripening mainly through repressing the expression of other genes rather than *FaLEA167*. In addition, in the *FaGAPC2-* and *FaPKc2.2*-overexpressed fruit, their expression levels were significantly increased, which largely inhibited strawberry fruit ripening, to prevent an infinite inhibition, the expression of the other negative regulators was thus reduced.

Fruit ripening is a complex process that involves substantive alterations in gene expression resulting changes in color, flavor, aroma and texture. Being one of the important indicators of stage of maturity, strawberry fruit color is formed due to anthocyanin accumulation [49], and represented by the color variables including lightness (L*), yellow to blue (b*) and green to red (a*). In the present study, OE of *FaLEA167* significantly reduced the content of total anthocyanins and a* value (Figs. 10A and F), confirming that *FaLEA167* may negatively regulated strawberry fruit ripening. Moreover, citric acid is regarded as the predominant organic acid in strawberry, accounting 49–75% of the total organic acid [24]. Its level could give an indication of degree of strawberry ripeness [50], and was suggested to decrease during fruit ripening of strawberry [51]. In our results, the citric acid content was found significantly increased by the OE of *FaLEA167* (Fig. 10E). The possible explanation is that *FaLEA167* OE inhibited strawberry fruit ripening, hence the transformed fruit conferred a lower level of ripeness and thus a higher level of citric acid. This may also explain the decrease of soluble protein and MDA content (Figs. 11A and B). It has been suggested that MDA and soluble protein increased as fruit ripen, the inhibition of fruit ripening by *FaLEA167* OE lead to less oxidative damage during fruit ripening, since MDA is a relevant standard to determine the oxidative force in plants [52]. The other ripening-related traits such as TSS, total sugar, phenolic and flavonoids content were not obviously affected by the OE of *FaLEA167*, indicating that they might not be the main attributors of the inhibition effects of fruit ripening by *FaLEA167* OE. However, how the *FaLEA* genes influence the ripening-related gene expression levels is still needed to be studied in the future.

## Conclusions

Taken together, a total of 266 FaLEAs were identified and characterized in strawberry genome. Among those, the members included in LEA1 and DHN groups were likely to function in fruit ripening, due to their larger proportion that differentially expressed during fruit development and ripening, and in the *FaGAPC2-* and *FaPKc2.2*-overexpressed materials. Transient overexpression of *FaLEA167* significantly reduced the percentage of fruit at full red stage but increased the amount of fruit at green stage, confirming its negative regulatory role in strawberry fruit ripening. This study gave a better understanding of *FaLEA* function in fruit ripening.

### Supplementary Information

**Additional file 1:** **Table S1.** Physicochemical characteristics of FaLEA proteins

**Additional file 2:** **Table S2.** Collinear pairs of FaLEA in cultivated strawberry, and among Arabidopsis and woodland strawberry.

**Additional file 3:** **Table S3.** The Ka, Ks and Ka/Ks values of FaLEA proteins in strawberry

**Additional file 4:** **Table S4.** Cis-elements number in FaLEA promoters

**Additional file 5:** **Table S5.** Differentially expressed FaLEA genes during fruit development and ripening

**Additional file 6:** **Table S6.** Primers used in this study

**Additional file 7:** **Fig. S1.** Chromosome location of FaLEA genes

**Additional file 8:** **Fig. S2.** Gene structures of FaLEA groups

**Additional file 9:** **Fig. S3.** Conserved in FaLEA proteins

## Data Availability

The RNAseq-based expression profiles were retrieved from the the data deposited in the CNGB nucleotide sequence archive (accession: CNP0002459 and CNP0004133), and NCBI SRA database (accession: PRJNA838938).
