# Supplementary figures and images for: Genome-wide analysis and functional validation reveal the role of late embryogenesis abundant genes in strawberry (Fragaria × ananassa) fruit ripening
Source: BMC Genomics. 2024 Mar 1;25:228. doi: 10.1186/s12864-024-10085-9 (PMC10908092; doi:10.1186/s12864-024-10085-9)

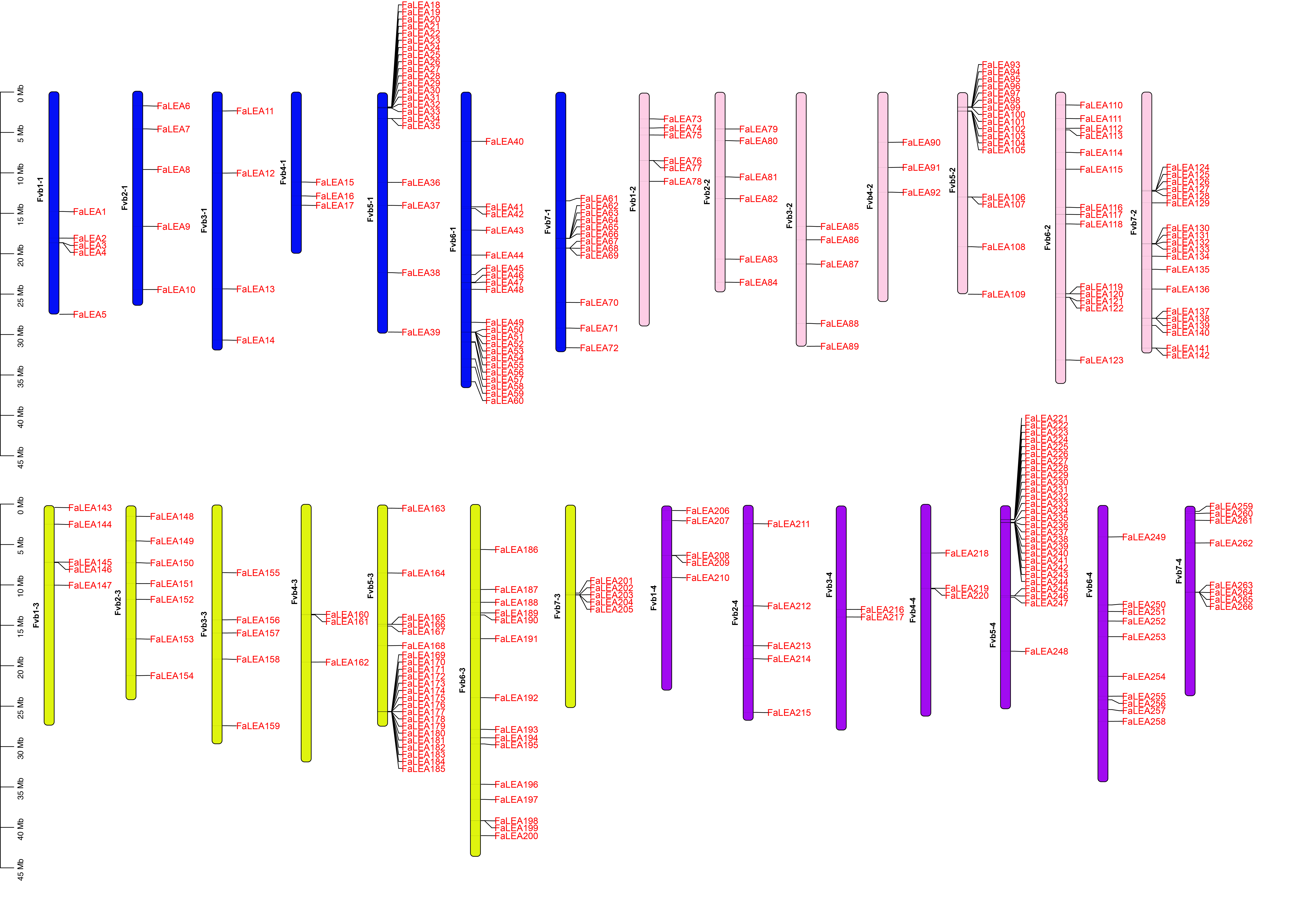

Supplement: Supplementary file 7 — Additional file 7: Fig. S1. Chromosome location of FaLEA genes [file 12864_2024_10085_MOESM7_ESM.jpg]
