# Supplementary material for: Genome-wide analysis and functional validation reveal the role of late embryogenesis abundant genes in strawberry (Fragaria × ananassa) fruit ripening
Source: BMC Genomics. 2024 Mar 1;25:228. doi: 10.1186/s12864-024-10085-9 (PMC10908092; doi:10.1186/s12864-024-10085-9)
Supplement: Supplementary file 8 — Additional file 8: Fig. S2. Gene structures of FaLEA groups [file 12864_2024_10085_MOESM8_ESM.pdf]

Figure S2. Gene structures of FaLEA groups.

LEA1 group:

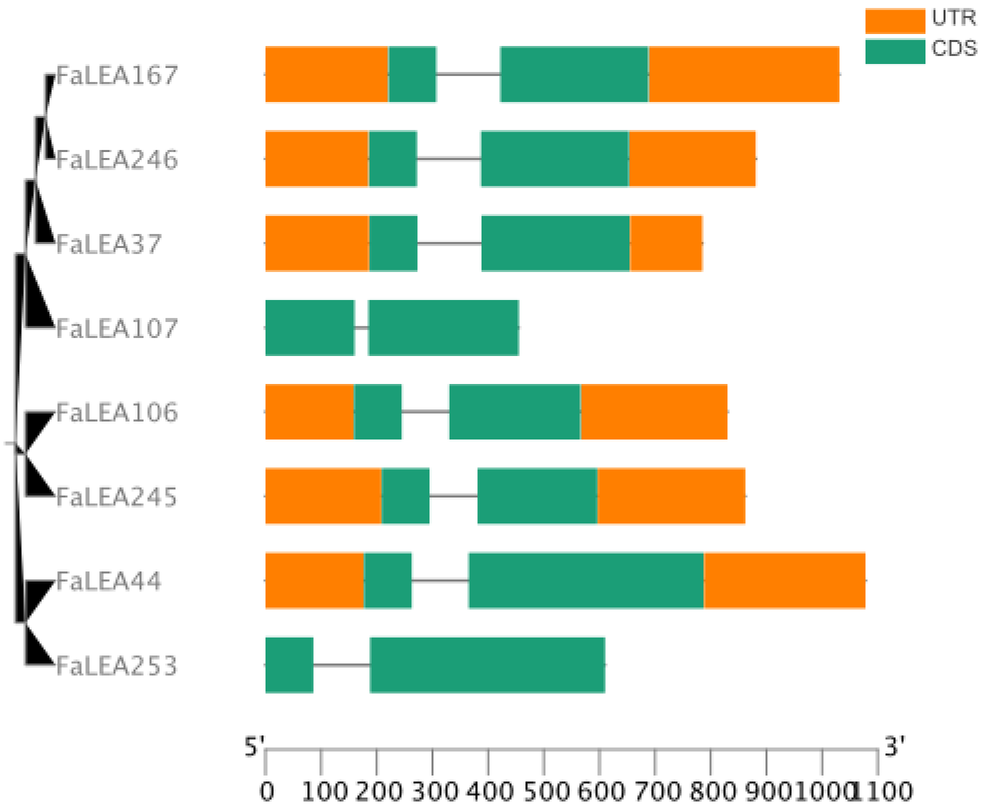

LEA2 group:

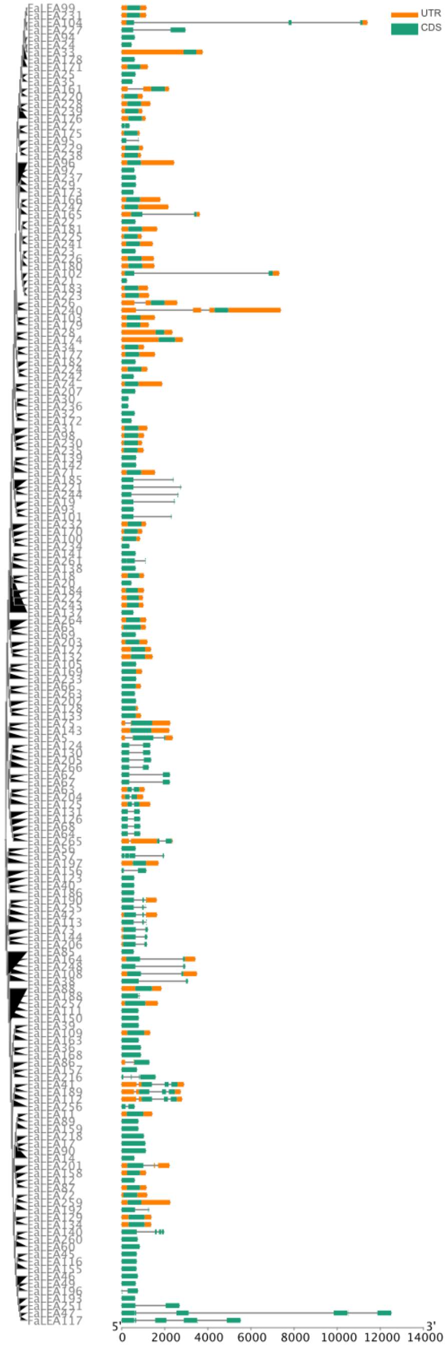

LEA3 group:

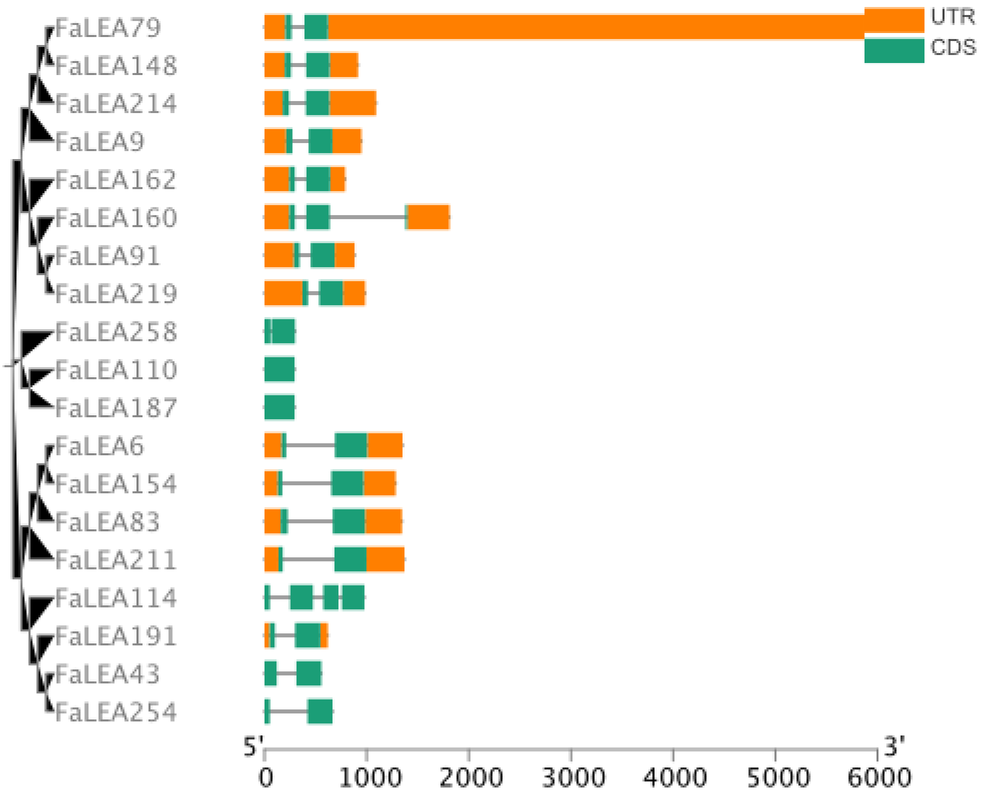

FaLEA79
FaLEA148
FaLEA214
FaLEA9
FaLEA162
FaLEA160
FaLEA91
FaLEA219
FaLEA258
FaLEA110
FaLEA187
FaLEA6
FaLEA154
FaLEA83
FaLEA211
FaLEA114
FaLEA191
FaLEA43
FaLEA254
UTR
CDS
5'
3'
0
1000
2000
3000
4000
5000
6000

LEA4 group:

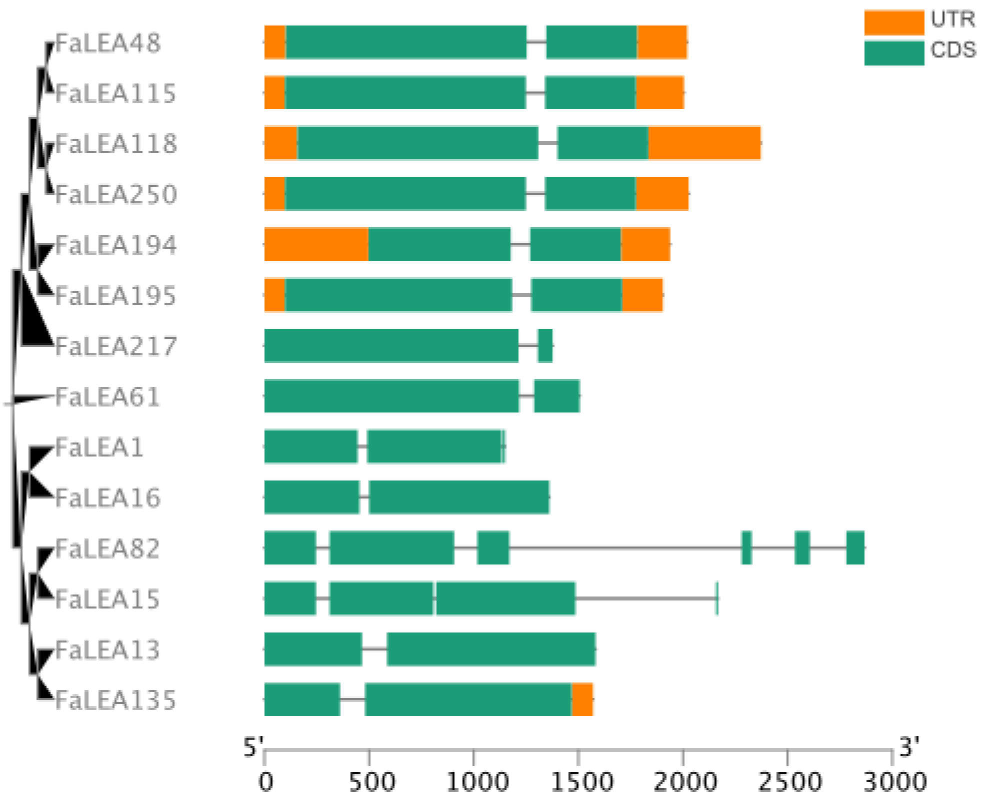

UTR
CDS
FaLEA48
FaLEA115
FaLEA118
FaLEA250
FaLEA194
FaLEA195
FaLEA217
FaLEA61
FaLEA1
FaLEA16
FaLEA82
FaLEA15
FaLEA13
FaLEA135
5'
3'
0
500
1000
1500
2000
2500
3000

LEA5 group:

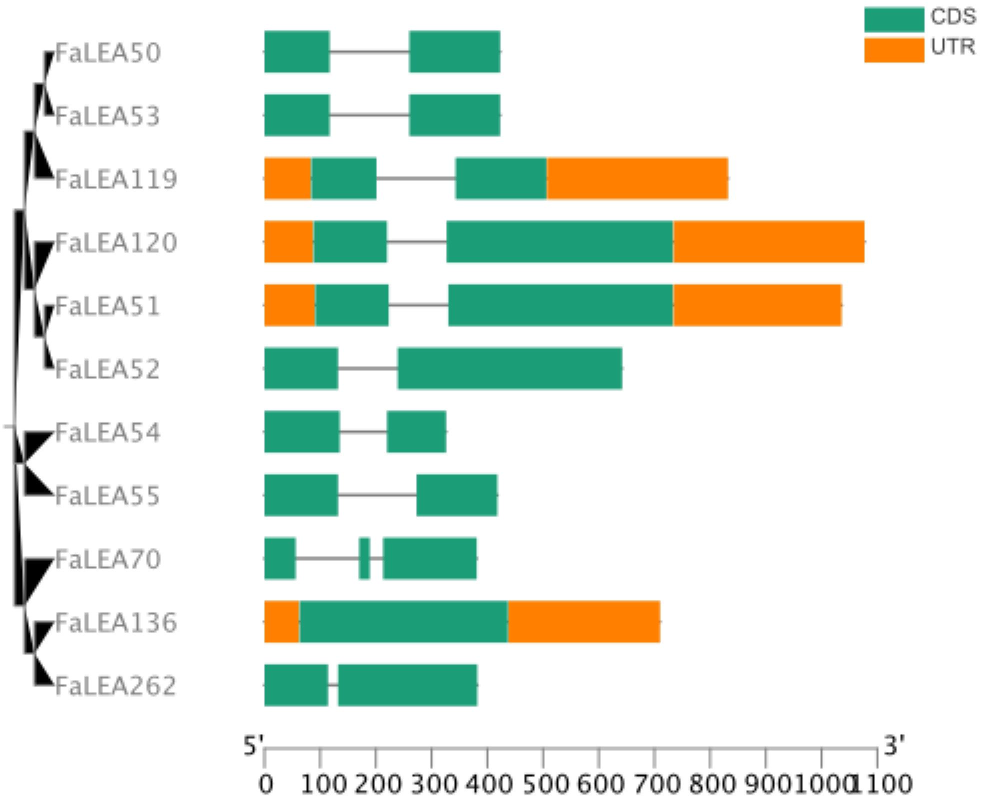

CDS
UTR
FaLEA50
FaLEA53
FaLEA119
FaLEA120
FaLEA51
FaLEA52
FaLEA54
FaLEA55
FaLEA70
FaLEA136
FaLEA262
5'
3'
0
100
200
300
400
500
600
700
800
900
1000
1100

LEA6 group:

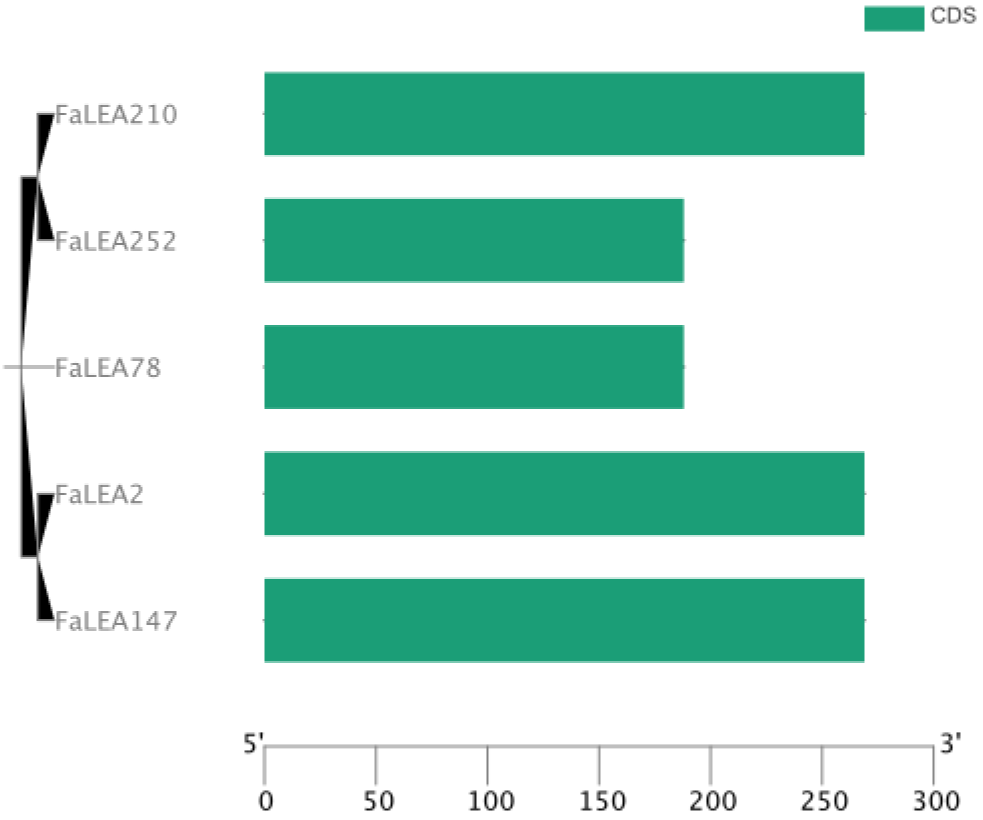
CDS
FaLEA210
FaLEA252
FaLEA78
FaLEA2
FaLEA147
5'
3'
0
50
100
150
200
250
300

DHN group:

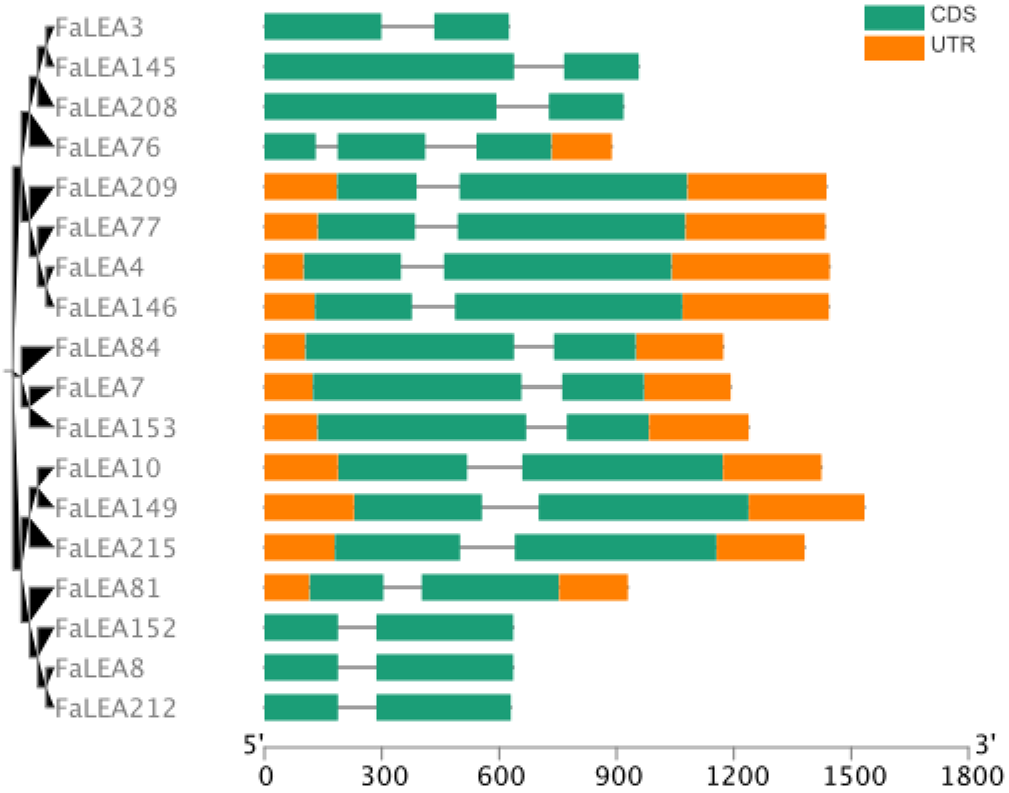

FaLEA3
FaLEA145
FaLEA208
FaLEA76
FaLEA209
FaLEA77
FaLEA4
FaLEA146
FaLEA84
FaLEA7
FaLEA153
FaLEA10
FaLEA149
FaLEA215
FaLEA81
FaLEA152
FaLEA8
FaLEA212
CDS
UTR
5'
3'
0
300
600
900
1200
1500
1800

SMP group:

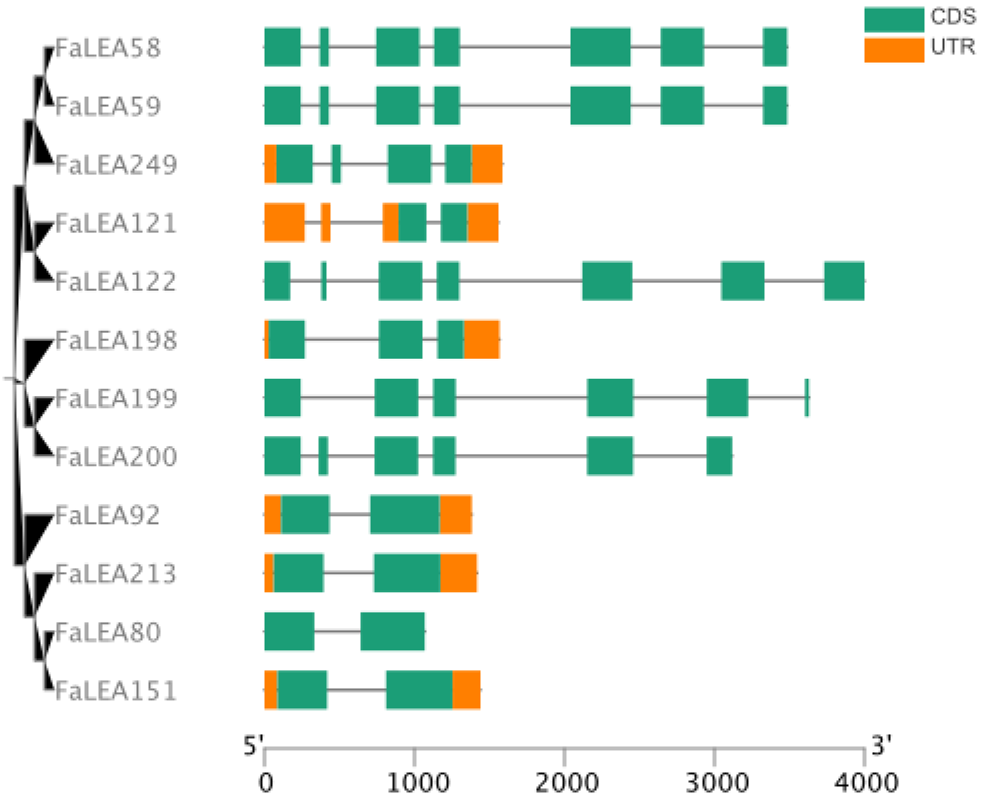

CDS
UTR
FaLEA58
FaLEA59
FaLEA249
FaLEA121
FaLEA122
FaLEA198
FaLEA199
FaLEA200
FaLEA92
FaLEA213
FaLEA80
FaLEA151
5'
3'
0
1000
2000
3000
4000
