# Supplementary material for: Genome-wide analysis and functional validation reveal the role of late embryogenesis abundant genes in strawberry (Fragaria × ananassa) fruit ripening
Source: BMC Genomics. 2024 Mar 1;25:228. doi: 10.1186/s12864-024-10085-9 (PMC10908092; doi:10.1186/s12864-024-10085-9)
Supplement: Supplementary file 9 — Additional file 9: Fig. S3. Conserved in FaLEA proteins [file 12864_2024_10085_MOESM9_ESM.pdf]

Figure S3. Conserved motifs in the FaLEA proteins.

LEA1 group:

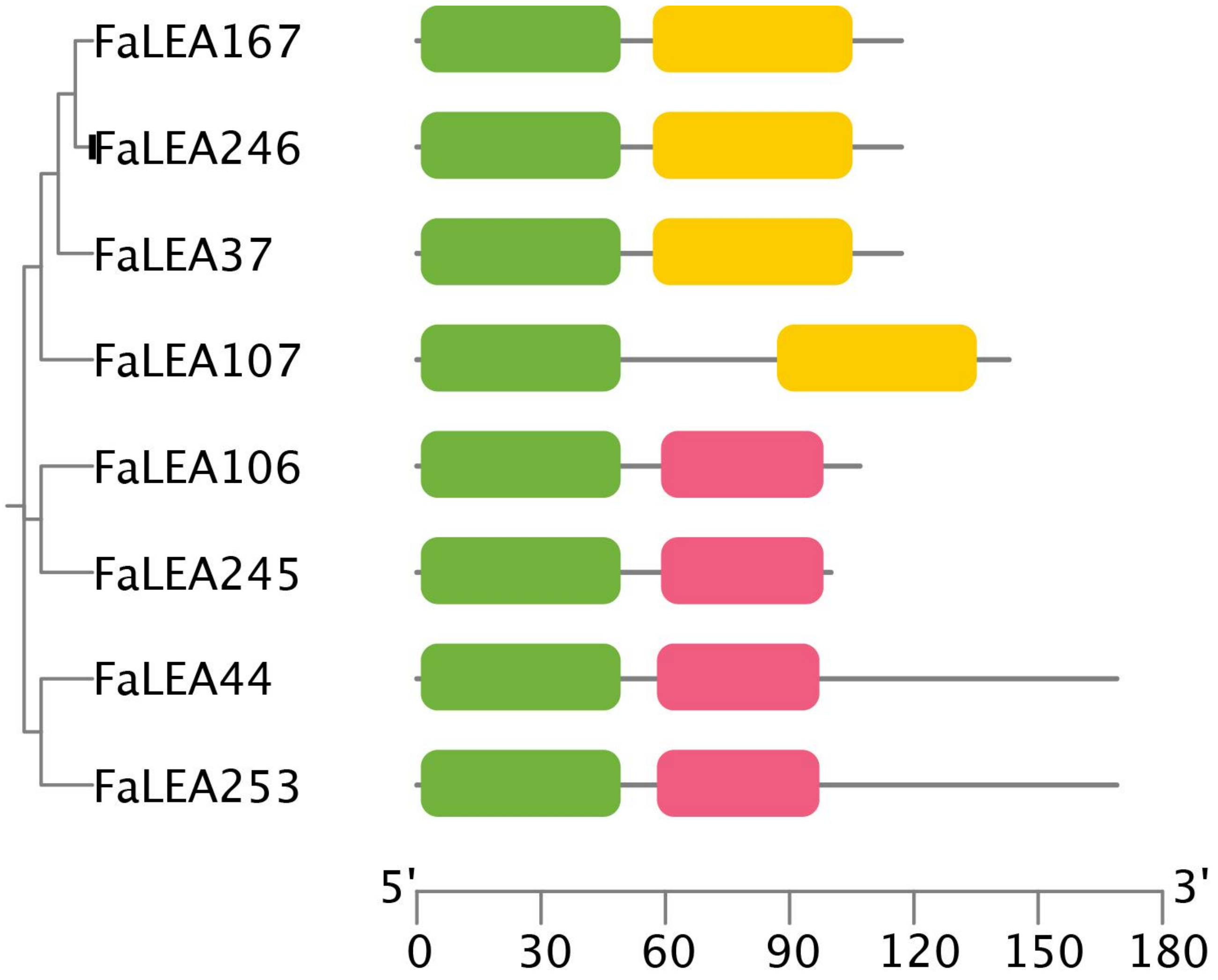

Motif	Symbol	Motif Consensus
1.		MQVAKEKLSNLASSAKEHIEIYKAKVAEKLEKARARTKEEKKMAKEKRKA
2.		MELHKAKARHAEKKLGAKLTHGHGIFNYHHPPPAPLHHHQHQHGHQHLGT
3.		QKEEKADHAADKKAGAAPAGSAPYGSGPPAYPLGGYGTRKL

LEA2 group:

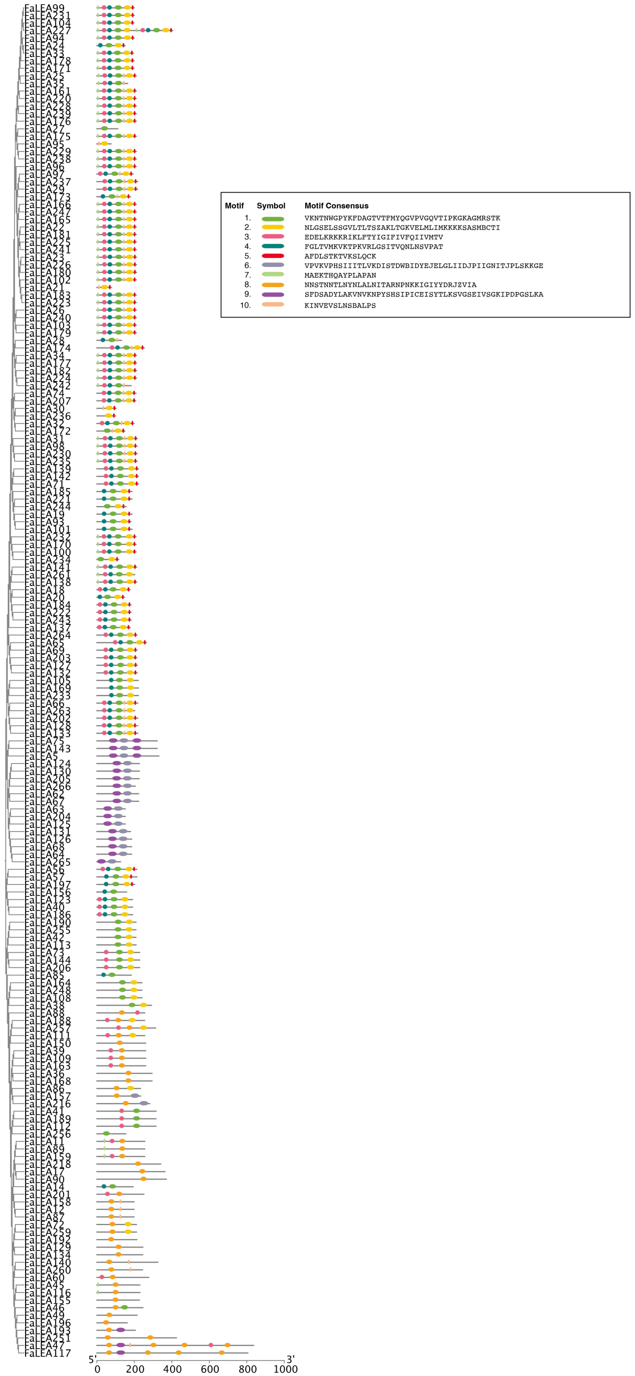
Motif Symbol Motif Consensus
1. VKNTNWGPYKFDAGTVTFMYQGVPVGQVTIPKGKAGMRSTK
2. NLGSELSSGVLTLTSZAKLTGKVELMLIMKKKKSASMBCTI
3. EDELKRKKRIKLFTYIGIFIVFQIIVMTV
4. FGLTVMKVKTPKVRLGSITVQNLNSVPAT
5. AFDLSTKTVKSLQCK
6. VPVKVPHSIIITLVKDISTDWBIDYEJELGLIIDJPIIGNITJPLSKKGE
7. MAEKTHQAYPLAPAN
8. NNSTNNTLNYNLALNITARNPNKKIGIYYDRJZVIA
9. SFDSADYLAKVNVKNPYSHSIPICEISYTLKSVGSEIVSGKIPDPGSLKA
10. KINVEVSLNSBALPS
5'
3'
0
200
400
600
800
1000

LEA3 group:

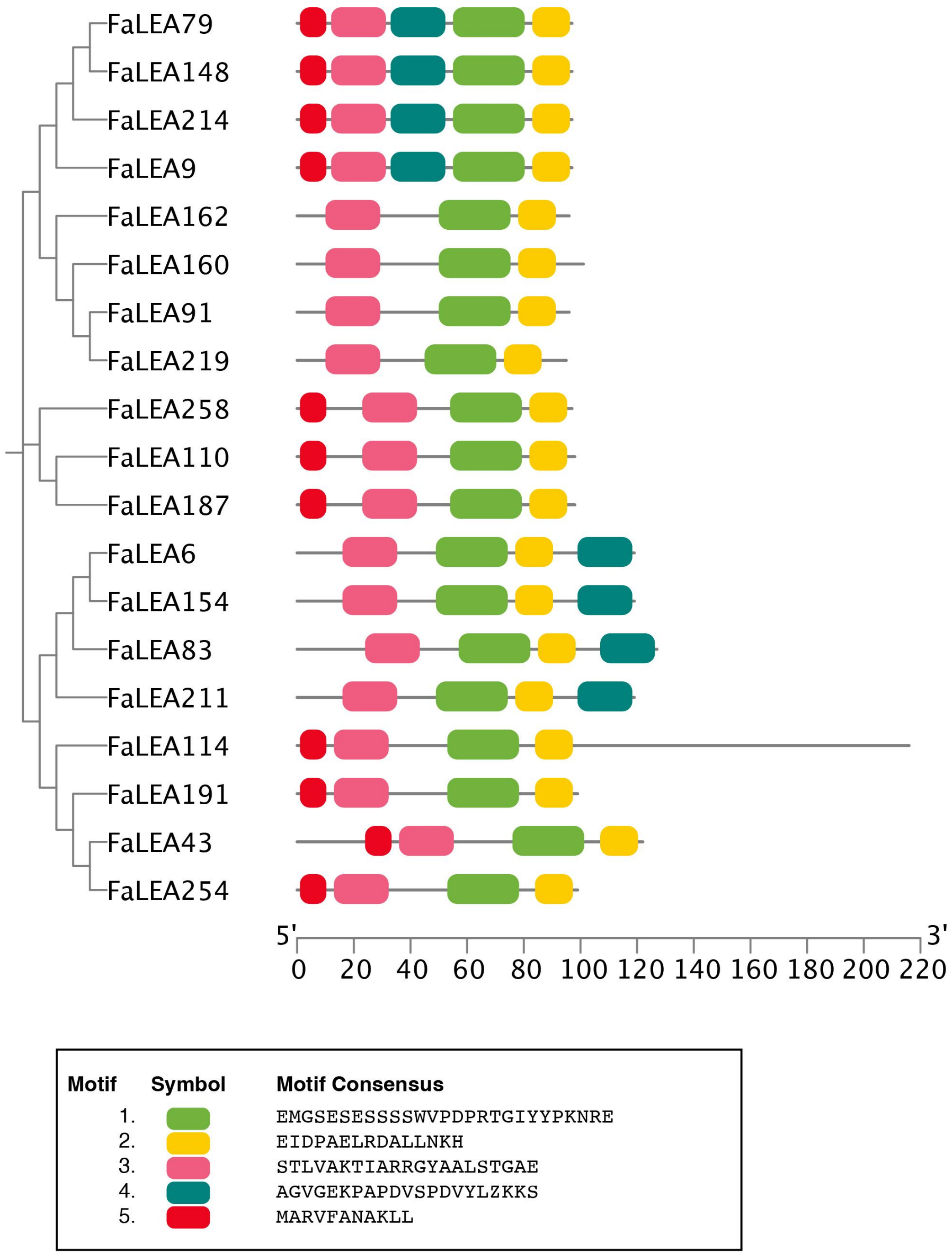
FaLEA79
FaLEA148
FaLEA214
FaLEA9
FaLEA162
FaLEA160
FaLEA91
FaLEA219
FaLEA258
FaLEA110
FaLEA187
FaLEA6
FaLEA154
FaLEA83
FaLEA211
FaLEA114
FaLEA191
FaLEA43
FaLEA254
5'
3'
0 20 40 60 80 100 120 140 160 180 200 220
Motif
Symbol
Motif Consensus
1. EMGSESESSSSWVPDPRTGIYYPKNRE
2. EIDPAELRDALLNKH
3. STLVAKTIARRGYAALSTGAE
4. AGVGEKPAPDVSPDVYLZKKS
5. MARVFANAKLL

LEA4 group:

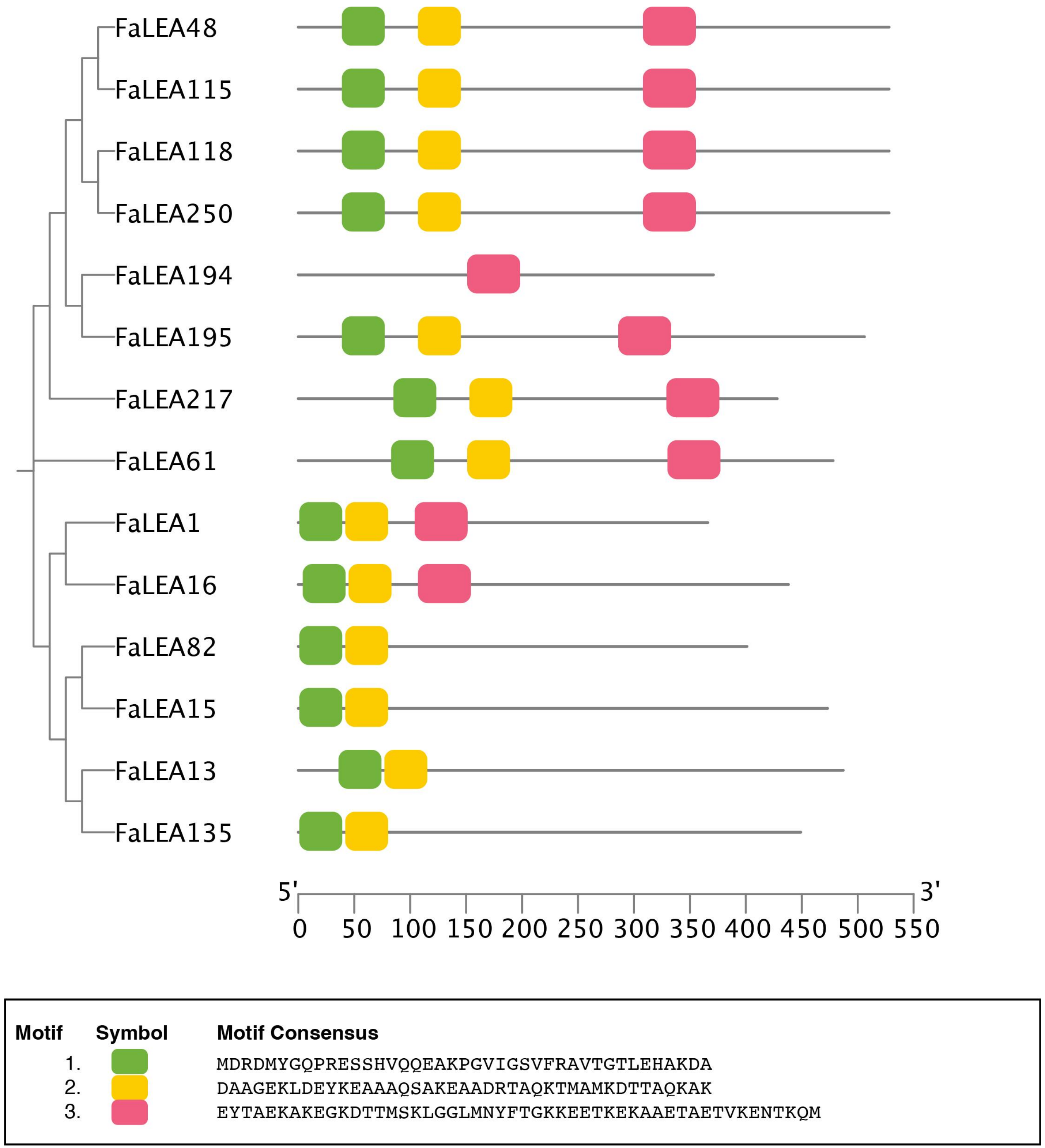
FaLEA48
FaLEA115
FaLEA118
FaLEA250
FaLEA194
FaLEA195
FaLEA217
FaLEA61
FaLEA1
FaLEA16
FaLEA82
FaLEA15
FaLEA13
FaLEA135
5'
3'
0 50 100 150 200 250 300 350 400 450 500 550
Motif
Symbol
Motif Consensus
1. MDRDMYGQPRESSHVQQEAKPGVIGSVFRAVTGTLEHAKDA
2. DAAGEKLDEYKEAAAQSAKEAADRTAQKTMAMKDTTAQKAK
3. EYTAEKAKEGKDTTMSKLGGLMNYFTGKKEETKEKAAETAETVKENTKQM

LEA5 group:

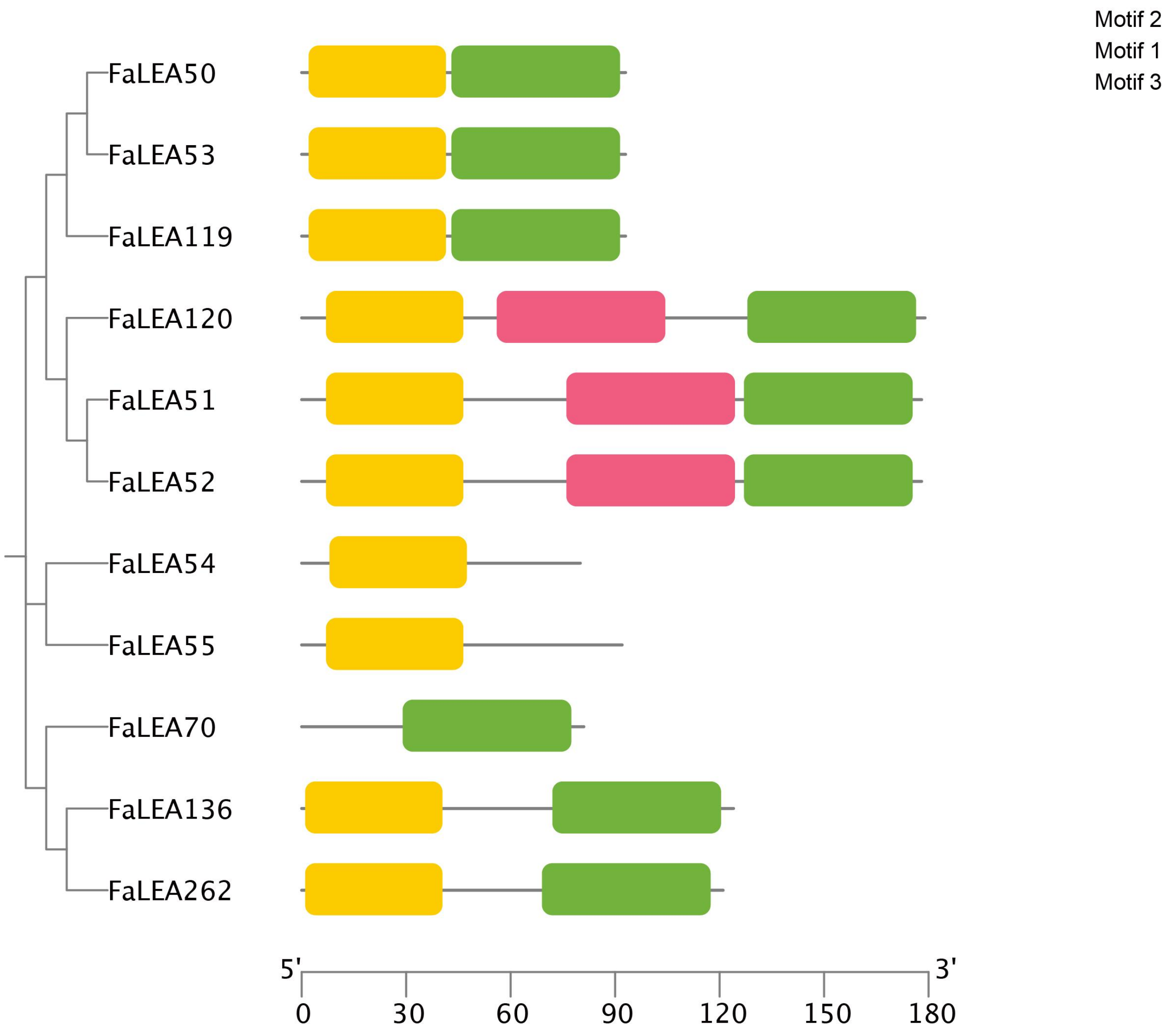

Motif	Symbol	Motif Consensus
1.	(green)	KGGETRKEQLGQEGYQEMGKKGGLSTTDKSGGERAAEEGIPIDESKYKTK
2.	(yellow)	RRDQZKREELDEKARQGETVVPGGTGGKSLKAQEHLAEGRH
3.	(pink)	QLGREGYQEIGHRGGETRKEQLGREGYQEIGHRGGETGKEQLGHEGYQQM

LEA6 group:

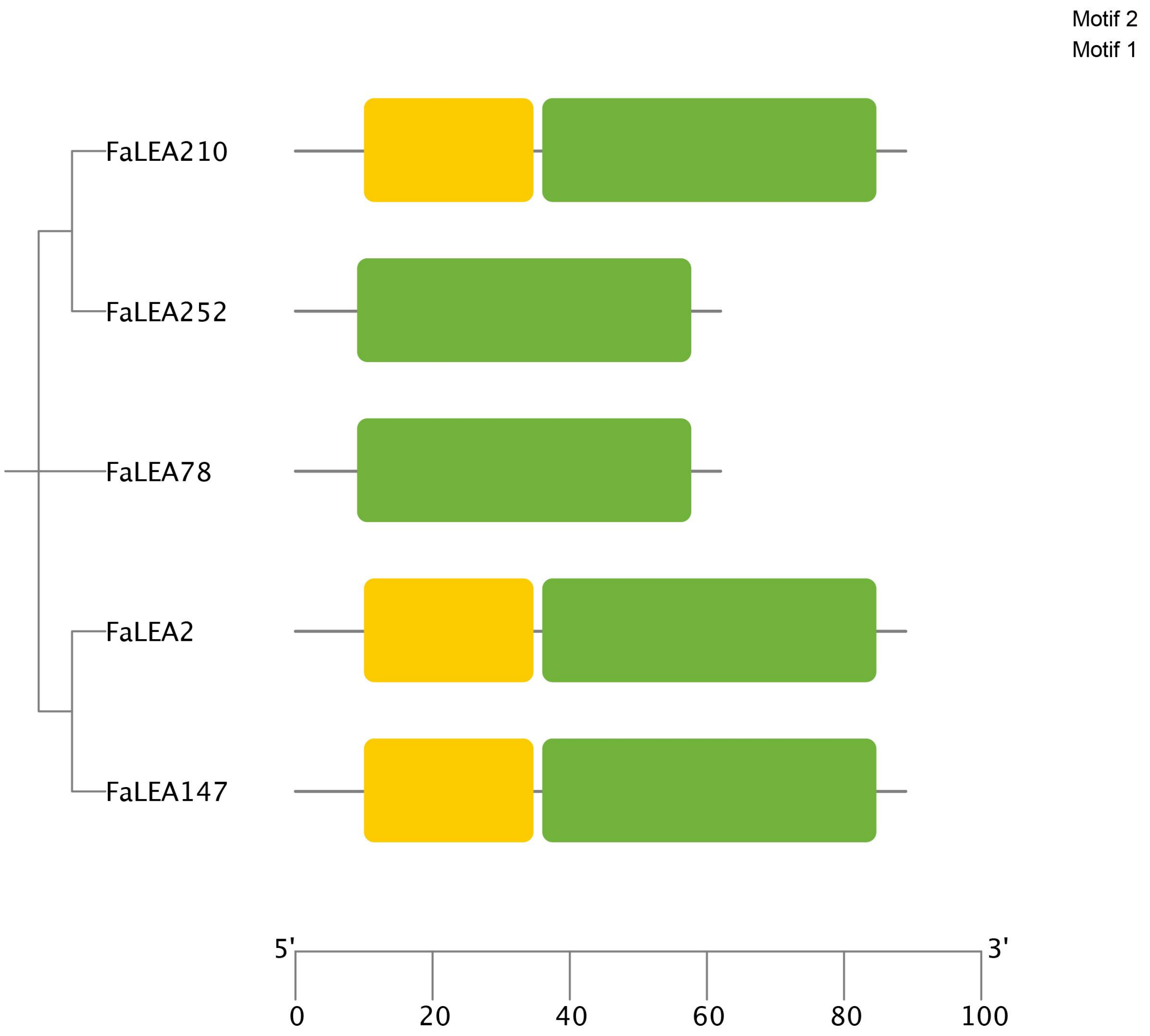

Motif	Symbol	Motif Consensus
1.	(green)	YDNLEDYKRQGYGTEGHQQVQPGRGPGATEGPTESGASKADVSATETINR
2.	(yellow)	EKKPAAAEKGSDAKLEQAMKDSPYLQ

DHN group:

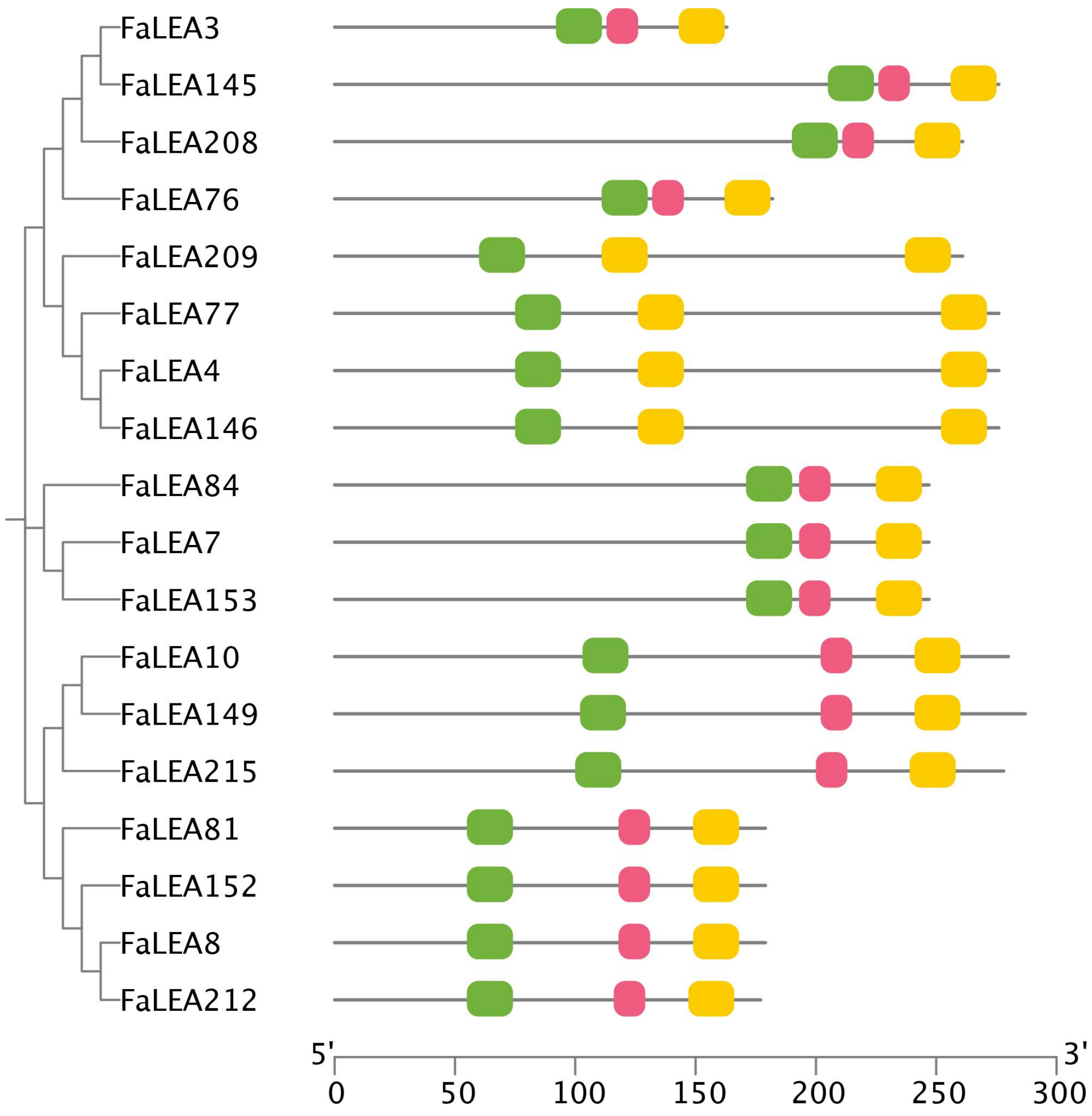

Motif	Symbol	Motif Consensus
1.	green	RHDSSSSSSSEDDGEGGRRKK
2.	yellow	GEQKEKKGMMEKIKEKLPGGH
3.	pink	KGLKEKIKEKLPGGH

SMP group:

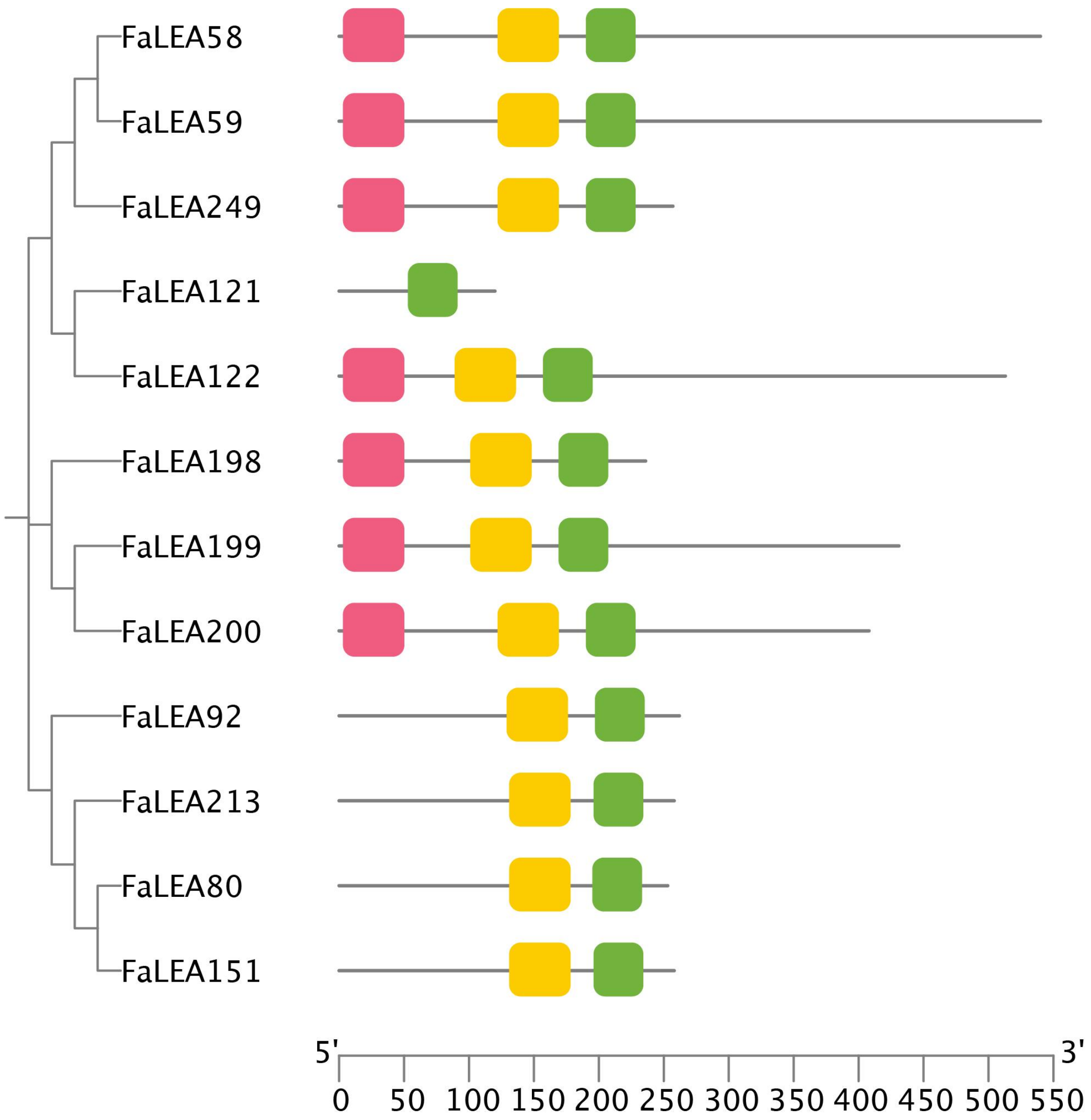

Motif	Symbol	Motif Consensus
1.	green	DKTKLADILADATSKLPADKPATRRDAEGVTGAEMRNDPYL
2.	yellow	GTGGQITIGEALEATAMTAGQKPVEWSDAAAIQAAEVRATGRTNIVPGGV
3.	pink	QEQPRRPVEDEQQKGGSQYGDIYLELAAEKAADSAVQSPVGLKLINSDHF
